# Zdhhc13-dependent Drp1 *S*-palmitoylation impacts brain bioenergetics, anxiety, coordination and motor skills

**DOI:** 10.1038/s41598-017-12889-0

**Published:** 2017-10-16

**Authors:** Eleonora Napoli, Gyu Song, Siming Liu, Alexsandra Espejo, Carlos J. Perez, Fernando Benavides, Cecilia Giulivi

**Affiliations:** 1 Department of Molecular Biosciences, School of Veterinary Medicine, University of California Davis, Davis, CA 95616 USA; 2 Department of Epigenetics and Molecular Carcinogenesis, The University of Texas MD Anderson Cancer Center, Smithville, and The University of Texas Graduate School of Biomedical Sciences at Houston, Houston, TX 77030 USA; 3 Medical Investigations of Neurodevelopmental Disorders (MIND) Institute, University of California Davis, Davis, CA 95817 USA

## Abstract

Protein *S*-palmitoylation is a reversible post-translational modification mediated by palmitoyl acyltransferase enzymes, a group of Zn^2+^-finger DHHC-domain-containing proteins (ZDHHC). Here, for the first time, we show that Zdhhc13 plays a key role in anxiety-related behaviors and motor function, as well as brain bioenergetics, in a mouse model (*luc*) carrying a spontaneous *Zdhhc13* recessive mutation. At 3 m of age, mutant mice displayed increased sensorimotor gating, anxiety, hypoactivity, and decreased motor coordination, compared to littermate controls. Loss of Zdhhc13 in cortex and cerebellum from 3- and 24 m old hetero- and homozygous male mutant mice resulted in lower levels of Drp1 *S-*palmitoylation accompanied by altered mitochondrial dynamics, increased glycolysis, glutaminolysis and lactic acidosis, and neurotransmitter imbalances. Employing *in vivo* and *in vitro* models, we identified that Zdhhc13-dependent Drp1 *S*-palmitoylation, which acting alone or in concert, enables the normal occurrence of the fission-fusion process. *In vitro* and *in vivo* direct Zdhhc13-Drp1 protein interaction was observed, confirming Drp1 as a substrate of Zdhhc13. Abnormal fission-fusion processes result in disrupted mitochondria morphology and distribution affecting not only mitochondrial ATP output but neurotransmission and integrity of synaptic structures in the brain, setting the basis for the behavioral abnormalities described in the *Zdhhc13*-deficient mice.

## Introduction

Protein *S*-palmitoylation is a reversible post-translational modification mediated by palmitoyl acyltransferase (PAT) enzymes, a group of Zn^2+^-finger, Cys-rich DHHC-domain-containing proteins [Zdhhc^1^]. The reversible addition of palmitate to Cys residues, by a labile thioester bond, is a dynamic process, unique among lipid modifications^1^. Palmitoylation increases the lipophilicity of proteins, influencing its subcellular distribution, stability, protein-protein interaction, trafficking, and function^1,2^. Some PATs are associated with abnormal cellular proliferation, particularly in several cancer models^3^ (and references therein).

Deficits in *Zdhhc17* (one of the 23 or 24 PATs from human and rat or mouse, respectively) are associated with behavioral, memory and synaptic defects^4,5^ based on the palmitoylation of substrates relevant to neurogenesis and neurotransmission (e.g. SNAP-25, synaptotagmin I, and huntingtin (Htt)^4,6^). Deficits in another PAT, Zdhhc13, were linked to alopecia, amyloidosis and osteoporosis^7,8^ and to behavioral deficits^5^ linked also to lower Htt palmitoylation^5,9^. To date, limited information is available on the functional connection between behavioral deficits and the substrates for these PATs.

Recently, we described the *luc* mice, carrying a naturally occurring recessive mutation on *Zdhhc13* ^3^. This loss-of-function allele (*Zdhhc13* ^*luc*^) was confirmed as a nonsense substitution, resulting in a premature stop codon (L203X)^3^. Phenotypically, homozygous *Zdhhc13* ^*luc*^/*Zdhhc13* ^*luc*^ (*luc/luc, luc*, homozygous or HOM) mice presented a generalized hypotrichosis with increased susceptibility to chemically induced skin carcinogenesis, whereas heterozygous (HET) mice had normal phenotypic appearance^3^. The *Zdhhc13* KO mice, developed by Dr. Hayden’s group, has a skin phenotype similar to the HOM mice and behavior consistent with Huntington’s disease (HD)^5^.

Given that (i) several mitochondrial proteins with clear roles in intermediary metabolism have been shown to be *S*-palmitoylated in a variety of biological systems^10–12^ and (ii) alopecia—as observed in our^3^ and another^12^ mouse model of Zdhhc13 deficiency—has been linked to mitochondrial dysfunction^13,14^, we hypothesized that *Zdhhc13* loss-of-function would result in deficits in bioenergetics, not necessarily limited to skin, but to other highly aerobic organs such as brain resulting in behavioral deficits associated with energy distress and altered metabolism of neurotransmitters. While several studies have reported complete *S*-palmitoylomes, very few have identified specific PAT substrates, the effect of this post-translational modification on the target’s function and the impact of this change at the whole organism level (behavior).

To this end, we evaluated the impact of Zdhhc13 deficiency on mitochondria activity and dynamics *in vitro* [HeLa and murine neuronal progenitor striatal cells (NPC)] and *in vivo* (cortex and cerebellum in 3- and 24-m old HET and HOM male mice) complemented by behavioral assessments (3-m old) of the C57BL/6NJ-*luc/luc* mice and littermate controls.

Our results indicated that Zdhhc13, acting via *S*-palmitoylation of Drp1, alone or acting in concert, is critical for sustaining cortex and cerebellum mitochondrial dynamics and function resulting in deficits in motor- and non-motor skills. This study, for the first time, shows that *S*-palmitoylation constitutes a critical component of the post-translational modifications of mitochondrial targets in the brain, affecting basic mitochondrial functions and behavior.

## Results

### Loss of Zdhhc13 results in behavioral deficits

**Figure 1:**
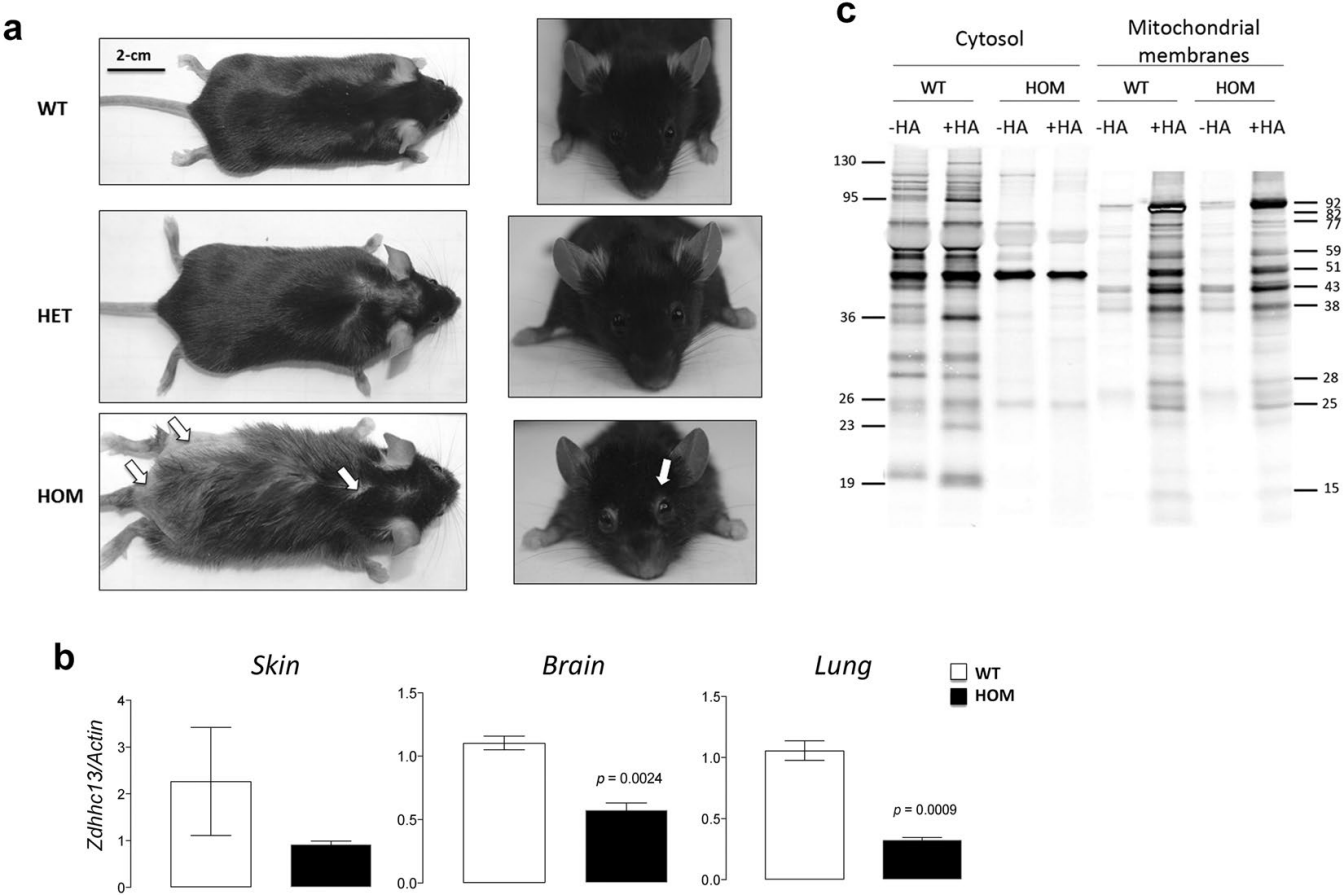
Skin phenotype, tissue gene expression and *S*-palmitoylation levels in Zdhhc13 deficiency. (**a**) Hair coat characteristics of *luc* mice. Pictures taken by Dr. F. Benavides. (**b**) *Zdhhc13* gene expression was performed by qPCR in skin, brain and lung from WT and *luc* mice. Statistical analyses were performed with Student’s *t* test. (**c**) *S*-palmitoylation detection in cytosolic and mitochondrial-membranes fractions from cerebellum of WT and HOM mice. Bars indicate proteins with decreased palmitoylation levels in HOM vs. WT. Molecular weights (in kD) were calculated with the Carestream software based on the MagicMark XP Western Protein Standard. Further details are reported in the Methods section. HA = hydroxylamine.

For this study, we generated a full-congenic C57BL/6NJ-*luc*/*luc* strain (N10) with the nonsense mutation in *Zdhhc13*. HOM mice are distinguished from WT littermates because of the generalized hypotrichosis and the characteristic lack of hair around their eyes. No apparent differences are observed between HET and WT mice (Fig. 1a). No other differences across genotypes were observed including the functional observation battery except for the baldness (Supplementary Figs S1–S2). For this study, only male mice were used to avoid confounding effects arising from estrous cycles.

Reduced *Zdhhc13* gene expression (>50%) was observed in skin, brain and lungs of HOM mice compared to WT (Fig. 1b). Zdhhc13-dependent protein *S*-palmitoylation was tested in cerebella from WT and HOM mice by using the acyl-biotin exchange assay (ABE; Fig. 1c). As expected for a nonsense mutation, a significant loss of palmitoylated proteins (annotated with lines and respective MW) was noted in both cytosolic and mitochondrial membrane fractions, supporting the role of Zdhhc13 as a PAT. These results opened the door for putative functional connections between this post-translational modification in brain mitochondria and their impact on both behavior and bioenergetics.

Deficiencies in Zdhhc13 have been linked to Huntington’s disease (HD) characterized by progressive neuropathology and motor deficits^5^. This inference was supported by the Zdhhc13-dependent *S*-palmitoylation of Htt and by Zdhhc17 and Zdhhc13 both being Htt-interacting proteins with dual functions as PATs and as favoring Mg^2+^ transport^6,9,15^. Given that the genetic background of our *luc* mice (C57BL/6NJ) is different than the *Zdhhc13* (Hip14l) KO mice utilized by Dr. Hayden’s group (FVB/N)^5^ and that genetic background affects the appearance of phenotypes even when carrying the same mutations^16^, behavioral tests (including those representative of the HD’s phenotype) were administered to both HOM and HET mice (3-m old). The tests were chosen to evaluate disease-specific and gene dose-dependent behaviors, including motor function, endurance, and gait [rotarod^17^, footprint analysis^18^]; learning and memory [fear conditioning^18^]; anxiety-related behavior [open field^17,19,20^]; reduction of muscle strength/hypotonia [grip strength^21^]; and schizophrenia/schizophrenia-like disorders [prepulse inhibition test^20^].

#### Motor deficits in *luc* mice

**Figure 2:**
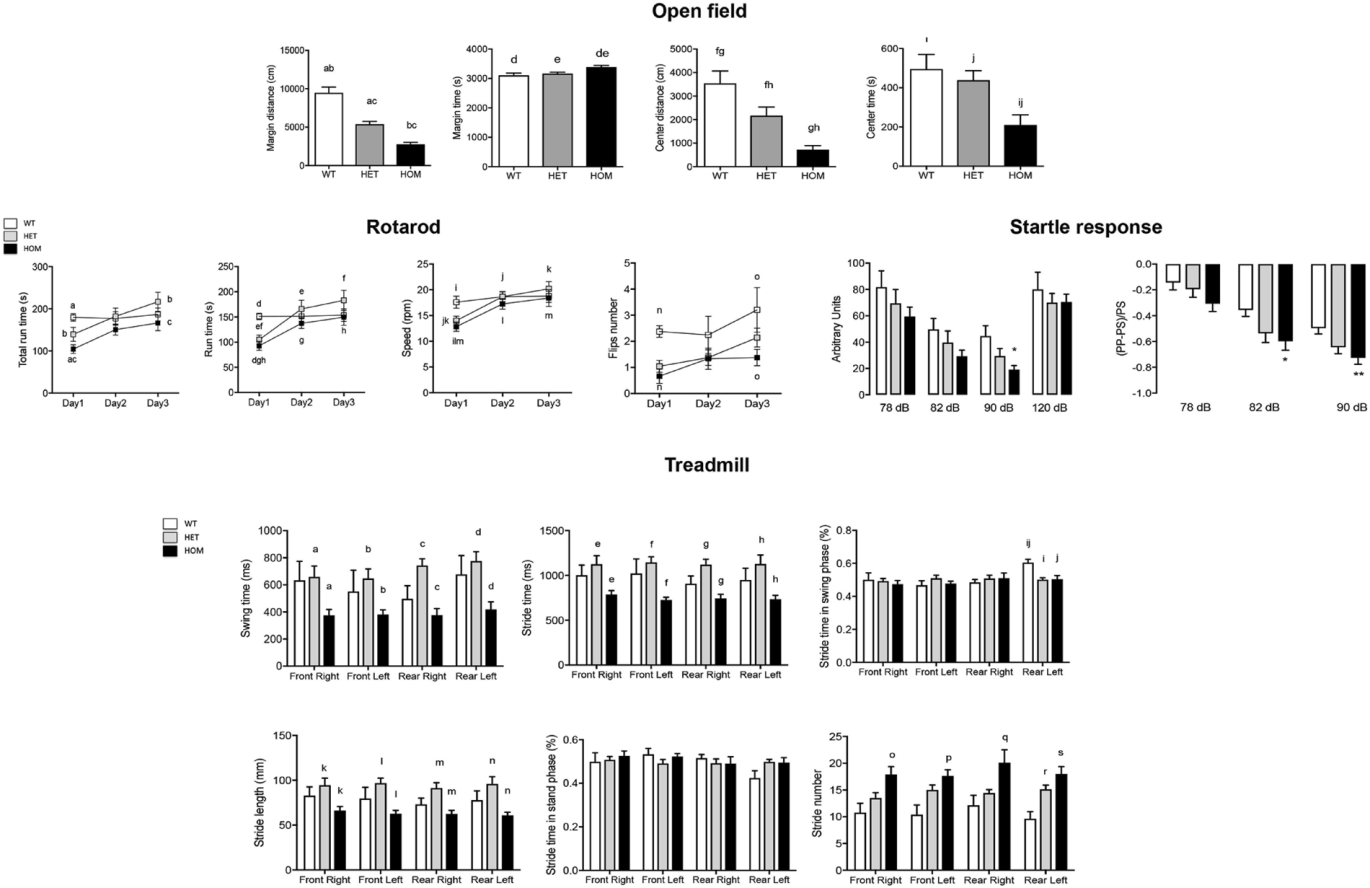
Evaluation of locomotor activity, coordination, gait and anxiety in *luc* mice. Representative outcomes are shown for 3-m old WT (*n* = 8), HET (*n* = 8) and HOM (*n* = 9; see Methods for details). All outcomes data are shown as mean ± SEM for each genotype and statistical analysis was performed by ANOVA followed by Bonferroni’s post-hoc test, unless otherwise noted. P values are reported right below each test. Other activities and locomotion parameters recorded are shown in Table 1 and Supplementary Fig. S2. P values are as follows. *Open field*: <0.0001 for a,b,g; 0.0021; 0.007 for d,i; 0.03 for e,j; 0.045 for f; 0.029 for h. *Rotarod*: Statistical analysis was performed with a 2-way ANOVA, using time and genotype as factors for the analysis, followed by Bonferroni’s post-test for multiple comparisons. P values are as follows: 0.0039 for a,b; 0.017 for c; 0.0009 for d; 0.0076 for e; 0.0005 for f; 0.0404 for g; 0.0064 for h; 0.013 for i; 0.030 for j; 0.0027 for k; 0.018 for l and o; 0.0022 for m; 0.021 for n. *Acoustic startle response*: *p = 0.014 **p = 0.0085 vs WT. *Treadmill*: Reported are outputs significantly different among the three groups. P values are as follows: 0.049 for a,b; 0.0063 for c; 0.018 for d; 0.036 for e; 0.045 for f; 0.0037 for g; 0.0302 for h; 0.0037 for i,j; 0.044 for k; 0.028 for l; 0.0082 for m; 0.019 for n; 0.0058 for o and p; 0.028 for q; 0.0097 for r; 0.0002 for s.

In the open field test, *luc* mice spent more time in the periphery and were overall less active (i.e., shorter distance traveled; Fig. 2). By evaluating spontaneous locomotor activity^22^ (Table 1), 14 (for HOM) and 8 (for HET) of the 17 outcomes tested confirmed hypoactivity and/or hypolocomotion relative to WT. The total distance traveled in the open field test showed a strong gene-dose dependency (R^2^ = 0.996; *p* = 0.040), with HET and HOM displaying significantly less travelled distance than WT (mean distance decreased by 30% and 70% of WT, respectively; *p* < 0.0001). Decreased stereotypic, horizontal and vertical movements were recorded in HOM only, whereas increased time spent stationary vs. mobile, as well as decreased number of clock- or counterclock-wise revolutions were significant in the *luc* mice relative to WT (Table 1).

**Table 1:** Measured outcomes for the assessment of spontaneous locomotor activity.

Outcome	WT	HET	HOM	ANOVA P values	R^2^	Regression P values
Total horizontal activity (counts)	12999 ± 1216^(a)^	13928 ± 858^(b)^	7862 ± 413^(a)(b)^	^(a)^0.0015 ^(b)^0.0003	0.618	0.424
Average horizontal activity (counts)	217 ± 20^(a)^	232 ± 14^(b)^	150 ± 11^(a)(b)^	^(a)^0.0041 ^(b)^0.0007	0.589	0.443
Total distance (cm)	13043 ± 1059^(a, b)^	7563 ± 671^(a, c)^	3155 ± 172^(b)(c)^	^(a)(b)^<0.0001 ^(c)^0.010	0.996	0.040
Total number of moves (counts)	641 ± 44^(a)^	711 ± 33^(b)^	329 ± 19^(a)(b)^	^(a)(b)^<0.0001	0.588	0.443
Moving time (s)	1584 ± 153^(a)(b)^	722 ± 63^(a)(c)^	339 ± 15^(b)(c)^	^(a)(b)^<0.0001 ^(c)^ 0.035	0.953	0.139
Rest time (s)	1373 ± 293^(a)(b)^	2878 ± 63^(a)^	3237 ± 28^(b)^	^(a)(b)^<0.0001	0.890	0.217
Vertical activity (rearing) (counts)	764 ± 122^(a)^	914 ± 87^(b)^	398 ± 65^(a)(b)^	^(a)^0.025 ^(b)^0.0012	0.475	0.516
Number of vertical moves (counts)	372 ± 31^(a)^	389 ± 29 ^(b)^	146 ± 16 ^(a)(b)^	^(a)(b)^<0.0001	0.694	0.373
Vertical time (s)	701 ± 101^(a)^	617 ± 37^(b)^	263 ± 35^(a)(b)^	^(a)(b)^<0.0001	0.888	0.218
Stereotypy (counts)	7554 ± 653^(a)^	6938 ± 468^(b)^	4359 ± 411^(a)(b)^	^(a)^0.0006 ^(b)^0.0045	0.890	0.217
Stereotypy time (s)	872 ± 34^(a)^	788 ± 19^(b)^	638 ± 36^(a)(b)^	^(a)^0.0003 ^(b)^0.0210	0.974	0.103
Clock-wise revolutions (counts)	87 ± 15^(a)(b)^	26 ± 5^(a)^	11 ± 1^(b)^	^(a)^0.0003 ^(b)^<0.0001	0.890	0.228
Counter-clockwise revolutions (counts)	95 ± 15^(a)(b)^	25 ± 2^(a)^	10 ± 1^(b)^	^(a)(b)^<0.0001	0.880	0.214
Left front quadrant time (s)	702 ± 166^(a)(b)^	189 ± 11^(a)^	147 ± 37^(b)^	^(a)^0.0070 ^(b)^0.0025	0.806	0.290
Right front quadrant time (s)	414 ± 78^(a)^	161 ± 6^(a)^	220 ± 66	^(a)^0.0070	0.537	0.476
Left rear quadrant time (s)	698 ± 125	534 ± 129	661 ± 223		0.05	0.862
Right rear quadrant time (s)	451 ± 50^(a)^	219 ± 36^(a)^	273 ± 66	^(a)^0.0170	0.538	0.476

Data are shown as mean ± SEM. n = 8 for WT and HET; n = 9 for HOM. Statistical analysis was performed by ANOVA followed by Bonferroni’s pairwise comparison post-hoc test. Counts: number of movements recorded by the beam for any given activity; Moving time: total time animal was moving; Rest time: Total time animal was not moving; Vertical time: Amount of time spent in vertical plane. Gene-dose dependency was evaluated for all the outcomes with a regression analysis built considering WT, HET and HOM as carrying respectively 0, 1 and 2 mutant alleles.

#### *Luc* mice show deficits in hind limb muscle strength

Deficits in muscle strength of hind- (not fore-) limbs assessed by the grip strength test were significant for HOM vs. WT (lower by 20%; *p* = 0.0063; Supplementary Fig. S2).

#### The treadscan (treadmill) analysis showed robust gait anomalies in *luc* mice

This test entails a thorough analysis of the animal’s gait when prompted to walk (as opposed to the open field which is self-initiated; Fig. 2). The number of strides was significantly higher (by 2-fold) in HOM mice accompanied by shorter stride distances (~50% of either WT or HET). The stride and swing times were shorter in HOM mice than WT and HET (by ~50%). These results indicated that HOM mice take more and shorter steps than WT or HET with no statistically significant differences in speed among the three genotypes (Supplementary Fig. S2).

For *luc* mice, the track width was longer for all 4 paws (by 20%) suggesting the need for a wider support base. The average print area was bigger for front and rear limbs on the left side (by 20%) with no differences for the right side. The significantly shorter limb swing times, broader track width with a broader average print area for left limbs in *luc* mice appear to stem from a balance deficit and/or trunk instability that is somehow compensated. Considering that spared white matter (after brain injury) correlates with various open-field and treadmill assessment measures in rodents (references therein^23^), these results may lend support that white matter-mediated behavioral functions may be negatively affected by Zdhhc13 deficiency similar to the hypomorphic Hip14-gt (gene-trapped) mouse with decreased Zdhhc17 expression in which deficits were observed in astrocytes and oligodendrocytes^11^.

#### *Luc* mice show coordination deficits but no learning or memory impairments

By utilizing a rotarod (gold standard test of motor coordination, fatigue resistance and balance in rodents), we evaluated latency to fall, total run time, and speed as indexes of motor activity, coordination and balance. A significant genotype effect was observed initially on time to fall from the rotarod or the speed at which mouse fell/flipped (HOM and HET; Fig. 2). Comparisons of the performance of mice over time showed that, in general, mutants showed intact motor skill learning capacity, albeit slower than WT, catching up with them by day 3.

No statistically significant genotype differences were recorded for the passive-avoidance/learning test (Fig. 2) indicating that the *luc* mice remembered which of a two-compartment chamber was paired with the aversive stimulus. Thus, the memory capacity and learning (this test and rotarod performance over time) were not impaired in *luc* mice.

#### *Luc* mice show anxiety-like behavior

The time spent in center area vs. the outer margin of the arena during the open field test was significantly shorter in HOM (by 63%; *p* = 0.05) than either HET or WT indicating increased anxiety-like behavior. A small, but significant effect on distance travelled in the center was observed for HOM only (decreased by 64% of WT and HET; *p* = 0.01).

#### Prepulse inhibition (PPI) of startle test shows increased sensory-gating activity in *luc* mice

While the results of the open field test may reflect increased anxiety, the observed hypoactivity or hypolocomotion of *luc* mice may confound this inference. Thus, a separate test for anxiety-like behaviors (i.e., PPI of startle test^24^) was utilized. In the PPI paradigm, a low-level acoustic stimulus (prepulse) is presented ms prior to a high intensity acoustic stimulus and whole body startle response is recorded. In healthy mammals, the prepulse significantly dampens the startle response to the pulse and the PPI is believed to reflect normal sensory gating of the CNS^25^. HOM mice showed a significant decrease in the maximum PPI, a basic marker of sensorimotor gating, consistent with increased anxiety and excitation of the cortico-striatal pathway (Fig. 2).

### Loss of Zdhhc13 results in a brain metabolic status consistent with a switch to aerobic glycolysis

Given the functional connection between anxiety^26^ and altered motor skills with mitochondrial dysfunction^27,28^, and the heavy reliance of brain on OXPHOS^29–31^, we tested brain bioenergetics by evaluating (a) mitochondrial Complex activities (along with citrate synthase as a surrogate marker for mitochondrial mass) in cerebellum and cortex and (b) cerebellar metabolomics. These regions were selected because *Zdhhc13* gene expression is higher in cerebellum than cortex (Supplementary Fig. S3) and some are implicated in anxiety disorders (cortex, amygdala, hippocampus, and striatum^26,32^), and motor skills and coordination (cerebellum, amydgala, hippocampus^33^). Finally, they can provide enough biological material for testing mitochondrial outcomes, and overall reduction in Zdhhc13 levels was assumed in all brain regions because this mouse model is not conditional or region-specific.

**Figure 3:**
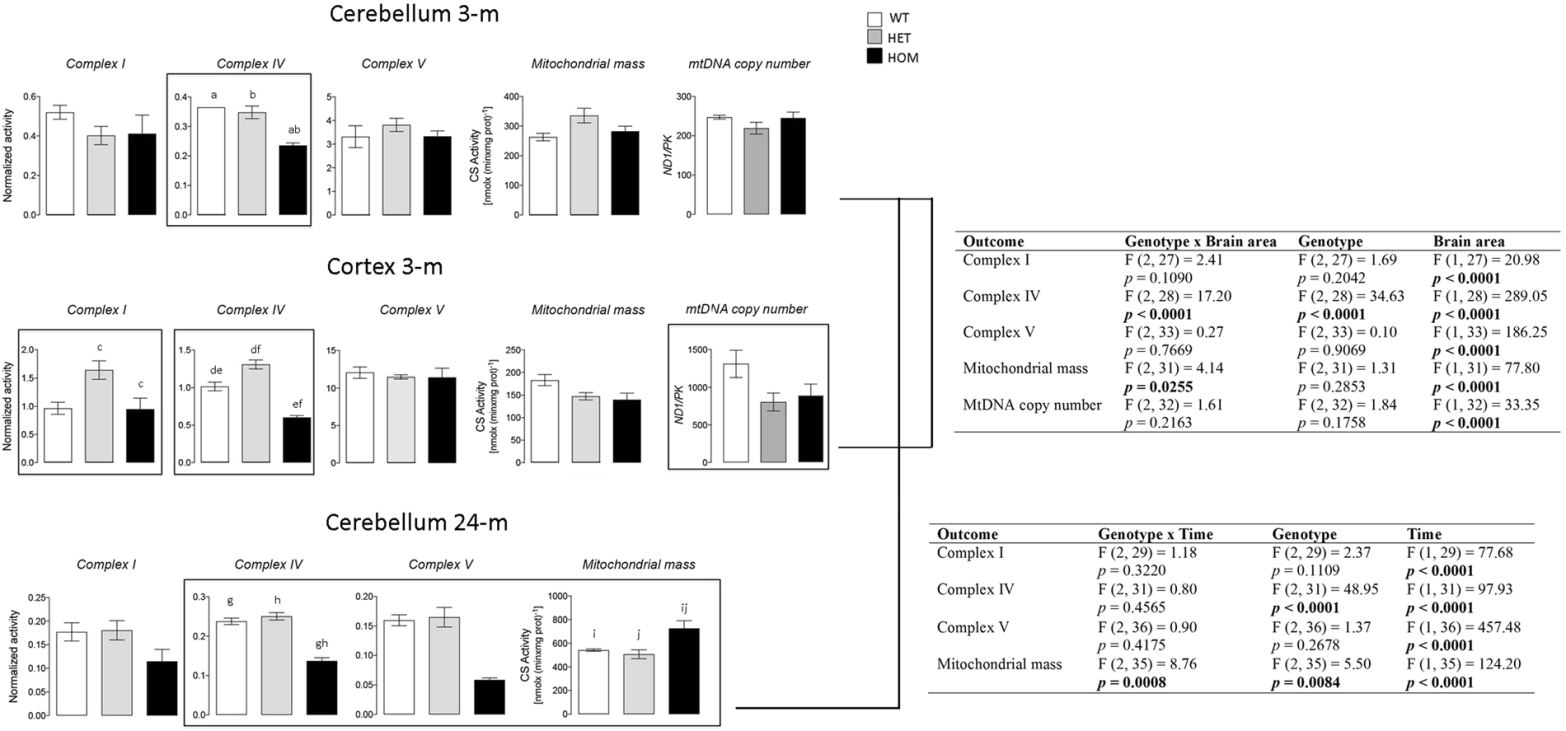
Effect of age and Zdhhc13 deficiency on brain mitochondrial outcomes. Cerebellar and cortical activities of Complex I, IV and V, expressed as nmol x (min x mg protein)^−1^, were normalized by citrate synthase activity. These outcomes were obtained from 3-m old WT (*n* = 3), HET (*n* = 8), HOM (*n* = 8) mice after the behavioral testing was performed, or from 2-y old WT (*n* = 12), HET (*n* = 6), HOM (*n* = 6). Statistical analysis was performed via 2-way ANOVA to evaluate the effect of genotype, time and their interaction on the total variance. In bold are statistically significant effects, and between parentheses are values of F (DFn, DFd). Bonferroni’s post-hoc test for multiple comparisons was also performed and statistically significant p values are as follows: a, b, e, f, g, h = <0.0001; c = 0.0102; d = 0.0014; i = 0.0003; j = 0.0002. All other details were included in the Methods section and Supplementary Methods.

Cerebella from 3-m old HOM mice showed a significant decline in normalized Complex IV activity (by 33%; Fig. 3), with no significant changes for Complex I, V, or citrate synthase activities and mitochondrial DNA copy number (mtDNA CN; Fig. 3). No significant differences were observed for any mitochondrial outcomes tested between WT and HET cerebella.

Similar to cerebellum, Complex IV activity was also decreased by almost half in cortex of HOM mice (40 and 53% relative to WT and HET, respectively; Fig. 3), whereas Complex I activity was significantly higher in HET (by ~2-fold) than HOM. Complex V activity and mitochondrial mass—as judged by the matrix biomarker citrate synthase—were not significantly different among the three groups. The mtDNA CN was significantly lower in both HET and HOM, without reaching the threshold for a mtDNA depletion. A 2-way ANOVA showed that brain area played a significant role in all the outcomes tested, whereas the genotype was mainly involved in the differences recorded for Complex IV. The genotype x brain area interaction influenced mainly Complex IV and mitochondrial mass (Fig. 3).

The effect of age on mitochondrial outcomes was tested by assessing Complex I, IV and V activities in cerebella of mice at 2 y of age (Fig. 3). The activity of Complex IV in HOM cerebella was almost 50% of that of either WT or HET whereas that of Complex V was nearly one-third. The deficits in these Complexes (normalized by mitochondrial mass) were magnified by the increased activity of citrate synthase (1.3-fold in HOM vs. HET or WT; Fig. 3) but not completely accounted for by this increase. A 2-way ANOVA revealed that age had the strongest effect on all outcomes tested (Fig. 3) whereas genotype influenced the most Complex IV activity and mitochondrial mass. The interaction between age and genotype was statistically significant only for mitochondrial mass (citrate synthase activity; Fig. 3).

**Figure 4:**
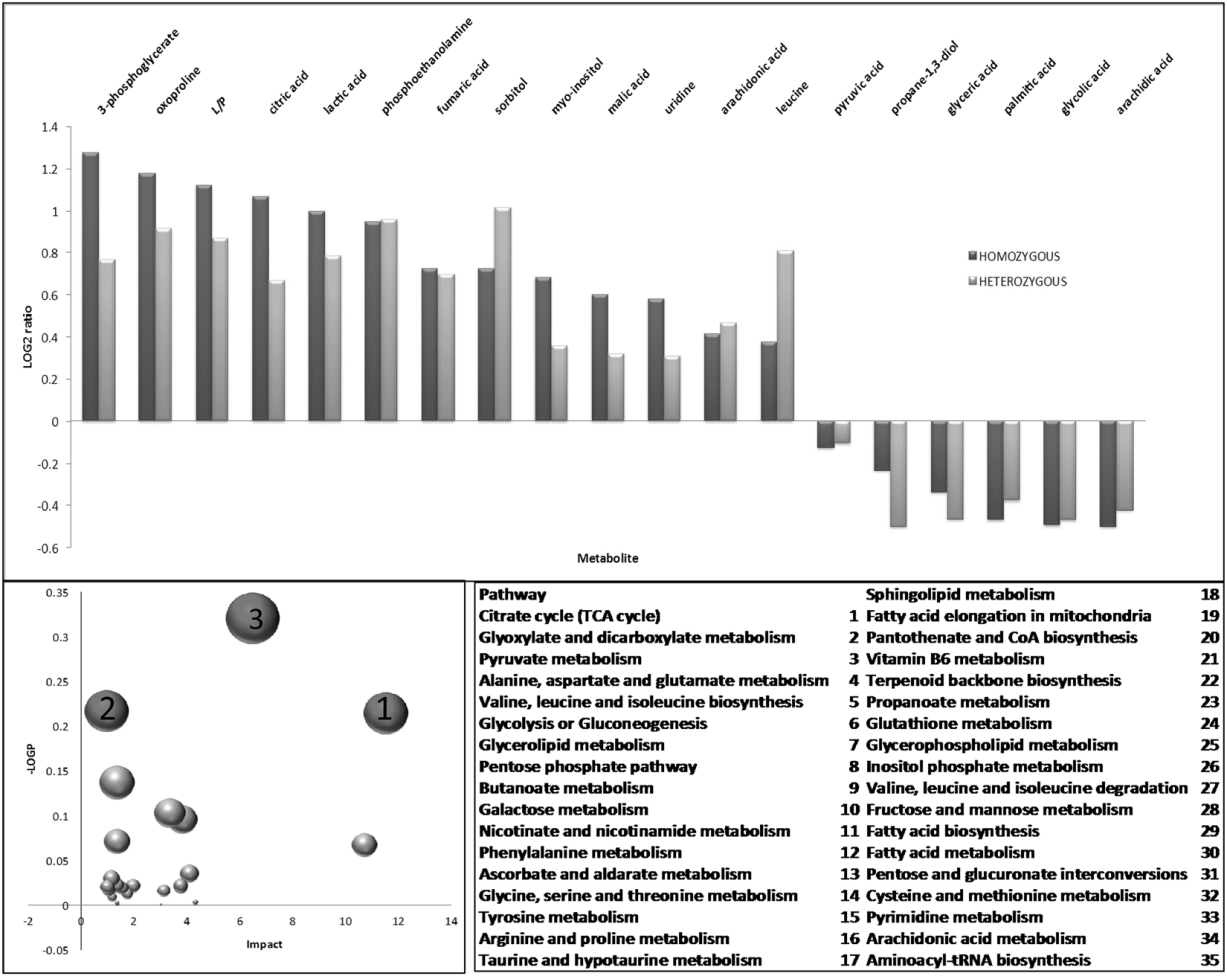
Untargeted metabolomics of cerebella from WT, HET and HOM mice. This analysis was performed in cerebella from 3-m old WT (*n* = 3), HET (*n* = 6) and HOM (*n* = 6) *luc* mice. Levels of metabolites statistically significantly different in at least one of the paired comparisons (WT-HET and/or WT-HOM) are shown. Lower panel: Pathway over-representation analysis was run with MetaboAnalyst. The size of the symbols is proportional to the impact. In dark gray, pathways with FDR < 0.05. The rest are indicated in light gray. The numbers were used to identify each of the pathways.

**Figure 5:**
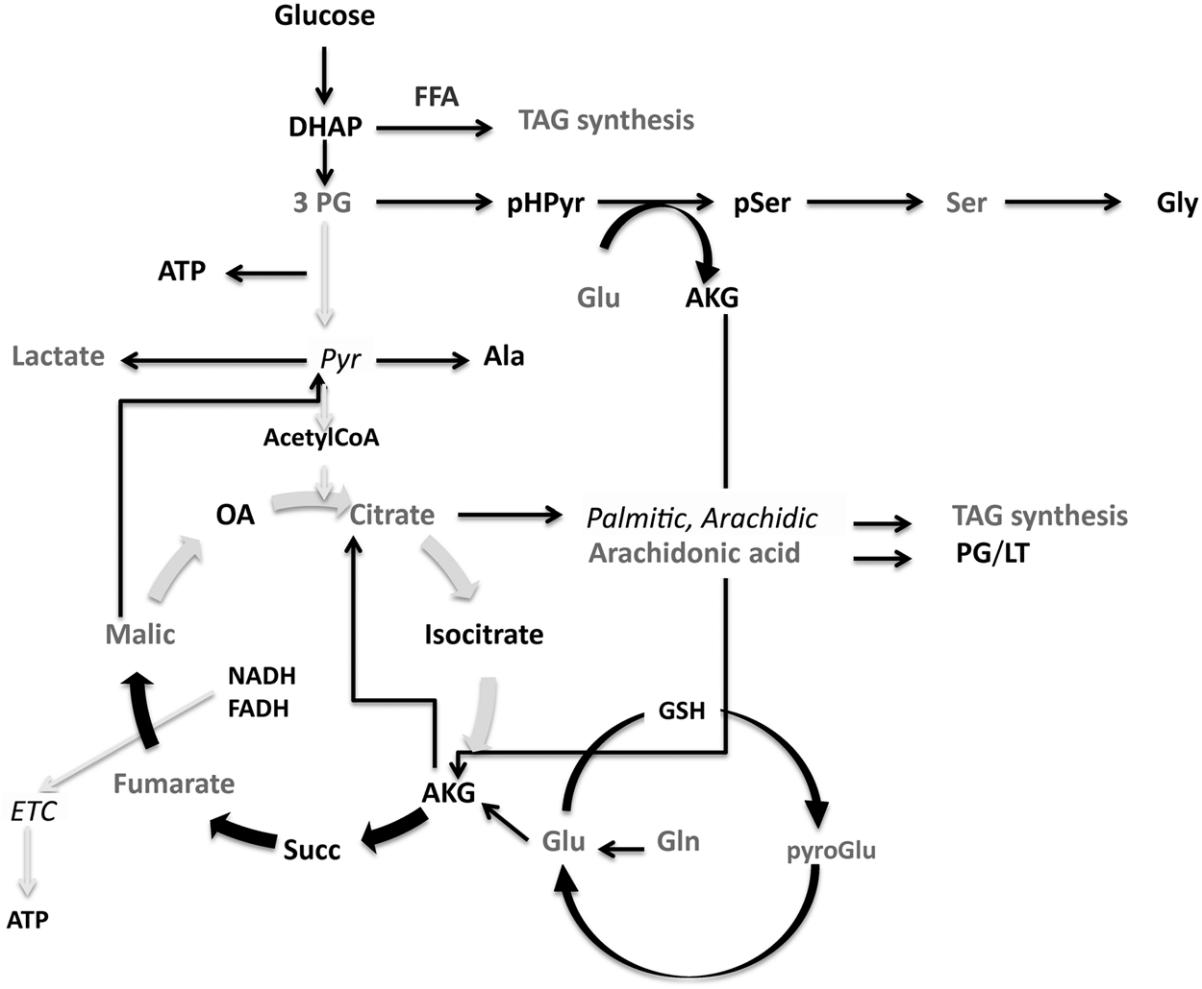
Scheme representing differences in metabolic flow of substrates in the presence of Zdhhc13 loss-of-function. Metabolites with differential levels in cerebella from WT and *luc* mice (from Fig. 4) are shown in light gray (higher abundance in *luc* than WT) and in italics (lower levels in *luc* than WT). Gray arrows indicate lower flux whereas black, enhanced pathways.

Considering the critical role of mitochondria in intermediary metabolism and neurotransmission, untargeted cerebellar metabolomics was performed to elucidate the role of the metabolic flow of substrates in the absence of Zdhhc13. A total of 214 metabolites were obtained, from which 121 were identified. From these, 18 were found statistically significantly different in at least one of the paired comparisons (WT-HET and/or WT-HOM; Fig. 4). The majority of these metabolites (67%) were more abundant in HOM cerebella than WT including the lactate-to-pyruvate (L/P) ratio. The increase in this ratio suggested a shift towards aerobic glycolysis. Using these 19 outcomes, pathway over-representation analysis was performed^34^, and from these, those with an FDR < 0.05 were selected (shown in red). The TCA cycle, glyoxylate, dicarboxylate and pyruvate metabolism were over-represented with Zdhhc13 deficiency (Fig. 4). Other pathways (with a raw *p* < 0.05 but with FDR > 0.05) included glycolysis, glycerolipid metabolism, pentose phosphate pathway, metabolism of several amino acids and ascorbate (Fig. 4, pathways 4–14). These metabolic changes are consistent with a shift from OXPHOS to glycolysis (increased L/P ratio, Fig. 4) accompanied by the use of both glucose and glutamine as fuels. While glucose generates ATP via glycolysis, this pathway also provides the synthesis of precursors for other biomolecules such as 3-phosphoglycerate for serine, glycine and alpha-ketoglutarate (AKG), and dihydroxyacetone phosphate (the latter used as the carbon skeleton for triacylglycerol synthesis). In this regard, the increased citrate concentration observed with Zdhhc13 deficiency seems to arise from increased reductive carboxylation of AKG to favor fatty acid synthesis (Fig. 5). The relatively lower levels of palmitic and arachidic acids seem to point to an increased triacylglyceride synthesis whereas increases in arachidonic acid may favor the production of prostaglandins and tromboxanes (Fig. 5). Taken together, these results show an increased anaerobic glycolysis likely to support biosynthetic pathways required for rapid cellular proliferation^35^, as well as support for synapse formation and growth^30^.

Remarkably, as the primary energetic costs in brain are associated with neurotransmission^29,31^, the increased levels of glutamate and glutamine in *luc* cerebella along with the higher levels of the γ-aminobutyric acid cyclization product, 2-pyrrolidinone (1.24-fold; *p* = 0.128 and 1.36-fold; *p* = 0.041 in HET and HOM of controls, respectively) are suggestive of a neurotransmitter imbalances, which could partly account for the abnormal behavior observed in *luc* mice.

### Pharmacological inhibition of Zdhhc13-dependent Drp1 *S*-palmitoylation halts mitochondrial targeting of Drp1 and alters mitochondria distribution

Remodeling of tubular mitochondria influences the internal diffusion of energy metabolites and proteins, the delivery of newly synthesized ATP to various cellular areas, the generation and maintenance of the electron protonmotive force, the stability of the respirasomes and the efficiency of OXPHOS^36^. Based on the following data obtained with *luc* mice (i) lower Complex IV activity accompanied by Complex V deficits observed with age likely as a result of accumulation of damaged mitochondria (Fig. 3), (ii) increased shift to aerobic glycolysis (Figs 4 and 5), and (iii) the lower *S*-palmitoylation of a band with MW matching that of Drp1 from *luc* brains (Fig. 1c), we hypothesized that Zdhhc13 deficiency altered the localization and/or activity of Drp1 enabling an altered fusion-fission process. Loss of Drp1 activity has been identified in several human neurological diseases^37–39^, with generalized OXPHOS deficiencies with or without lower cytochrome *c* oxidase activity^39–41^. Drp1 deficiency is generally thought to cause mitochondrial dysfunction due to a failure of a Drp1-dependent mechanism of mitophagy that removes damaged mitochondria^42^ in line with the worsening of bioenergetics with age. While two studies reported *S*-palmitoylation of Drp1^10,43^, no functional consequences for this modification were reported.

**Figure 6:**
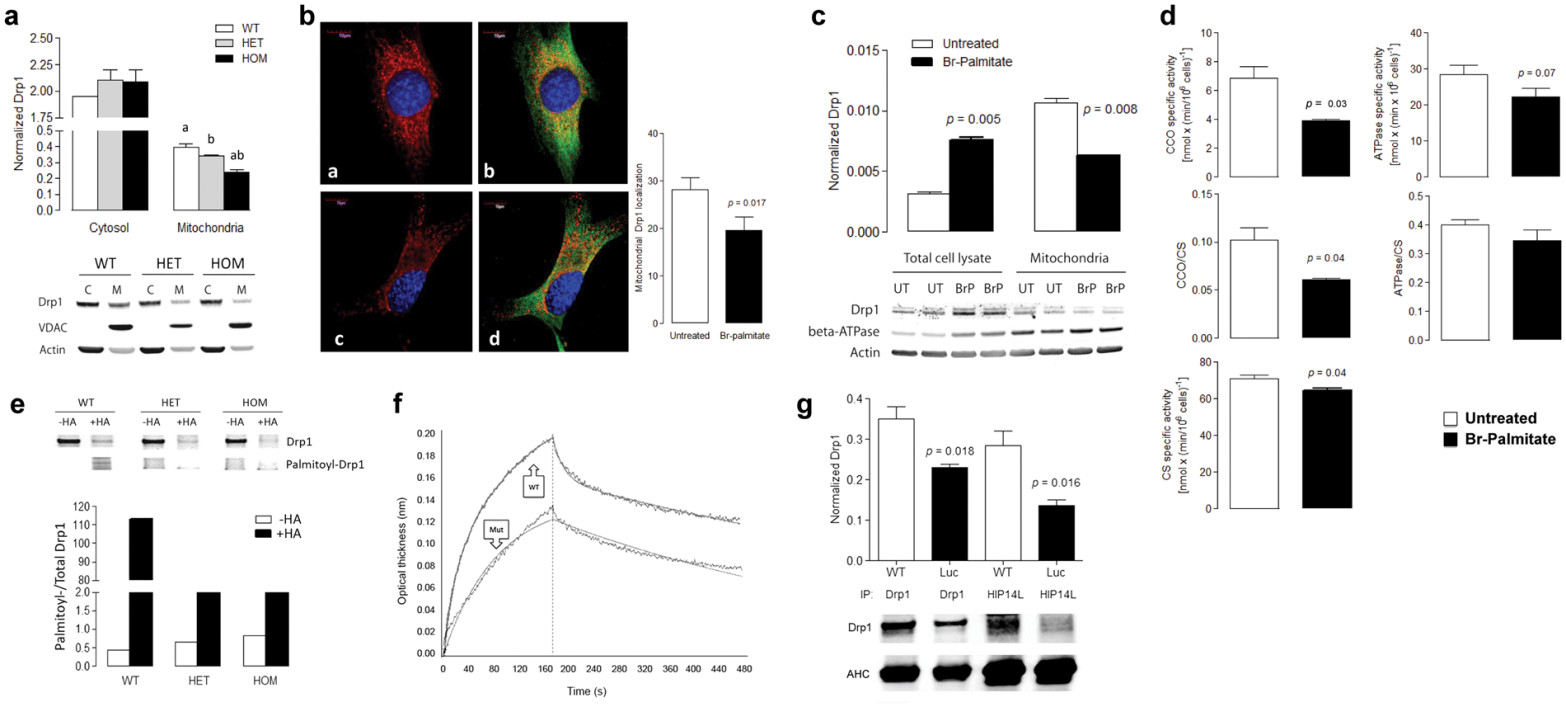
Evidence of the direct protein interaction between Zdhhc13 and Drp1. (**a**) Representative cropped image of Drp1, VDAC and actin levels in cytosolic and mitochondrial cerebellar fractions from WT, HET and HOM mice (*n* = 3). Data are reported as mean ± SEM. Statistical analysis was performed by ANOVA, followed by Bonferroni’s post-hoc test. P values are as follows: a = 0.015; b = 0.049. (**b**) Mitochondria (red) morphology, distribution and mass, and Drp1 levels (green) in NPC vehicle-treated (a,b) and treated (c,d) with 20 µM 2-bromo-palmitate for 6 h. Pictures were taken at a 120x magnification. Mitochondrial Drp1 levels were evaluated using the Image J (Fiji) colocalization plugin and reported as mean ± SEM of ≥10 fields. Statistical analysis was performed with paired Student’s t test. (**c**) Drp1, beta-subunit of mitochondrial ATPase and actin levels in total and mitochondrial fractions of NPC before and after treatment with 20 µM 2-bromo-palmitate for 6 h. Densitometry was evaluated with the Carestream software and data are reported as mean ± SEM of 2 separate experiments. Statistical analysis performed with paired Student’s t test. (**d**) Complex IV and V activities are shown as either normalized or non-normalized by citrate synthase. Specific activities are expressed as nmol x (min × 10^6^ cells)^−1^. Statistical analysis performed by paired Student’s *t* test between vehicle-treated and bromo-palmitate-treated samples. (**e**) Cerebellar Drp1 palmitoylation levels (cropped images). Immunoprecipitation of Drp1 was followed by ABE assay (see Methods). Levels of *S*-palmitoylated Drp1 normalized to total Drp1 are reported. HA = hydroxylamine. (**f**) Interaction of purified, recombinant Drp1 with full length and truncated GST-tagged Zdhhc13. The kinetics traces were obtained with WT and mutant Zdhhc13 at concentrations indicated in the text. Other details, see Methods. (**g**) Representative cropped image showing Drp1 levels in WT and HOM brains upon immunoprecipitation with anti- Drp1 or Zdhhc13 antibodies. Densitometry analysis was performed with Image J. The antibody heavy chain was used as loading control. Statistical analysis was performed with the Student’s *t* test. Representative full-length western blot images are shown in Supplementary Fig. S5.

Mitochondrial and cytosolic fractions from cerebella of WT and HOM mice were used to test for the Zdhhc13-mediated subcellular distribution of Drp1. A negligible cytosolic contamination was evidenced by the low levels of actin in the mitochondrial fractions and the effectiveness of mitochondria extraction by the lack of Vdac detection in the cytosolic fractions (Fig. 6a). Purity of these fractions was supported by the 30 to 300-fold enrichment of mitochondrial proteins in the mitochondria fraction (Supplementary Table S1). Levels of cerebellar Drp1 from WT and HOM mice, as judged by western blots, was not different in the cytosol (normalized by actin) but showed a significant decrease in the mitochondrial fraction (normalized to Vdac) of the HOM mice (~50% of WT; Fig. 6a).

Consistent with these results, specific pharmacological inhibition of *S*-palmitoylation in NPC obtained with exposure to 20 µM 2-bromo-palmitate^15^ for 6 h, decreased significantly the co-localization of Drp1 with mitochondria by ~50% both in confocal (Fig. 6b) and immunoblotting experiments (Fig. 6c). As observed with the younger mice, normalized Complex IV activity in 2-bromopalmitate-treated cells was lower than vehicle-treated (by ~50%) whereas normalized Complex V activity was not affected (Fig. 6d). Of note, mitochondria morphology, distribution and mass (visualized by using MitoTracker Red, which specifically stains polarized mitochondria) in 2-bromo-palmitate- vs. vehicle-treated NPC (Fig. 6b; compare subpanels a and c) showed a significant decline in functional mitochondria (normalized per cell area; *p* = 0.035), accompanied by increased perikarya clustering compared to the more evenly distributed mitochondrial network in vehicle-treated NPC. This pattern was similar to mammalian systems with loss of Drp1 in which disrupted morphology (depending on cell type^44,45^), nuclear clustering, mitochondria hyperdensity and increased spheres/globules^46,47^ was reported.

Similar results and consistent with this idea, L203X Zdhhc13-overexpressing HeLa cells relative to WT Zdhhc13-overexpressing cells had lower polarized mitochondrial mass (by 21%), lower Drp1 in mitochondrial foci (by 23% normalized to polarized mitochondria; *p* = 0.044; Supplementary Figure S4), with a more compact mitochondrial distribution, possibly due to the presence of a hyperfused mitochondrial network, with no differences in the amount of total (cellular) Drp1 between groups (Supplementary Results and Supplementary Fig. S4).

Thus, inhibition of Drp1 *S*-palmitoylation or overexpression of L203X Zdhhc13 results in a significant altered mitochondria distribution extending the results obtained *in vivo*.

### Zdhhc13 interacts with Drp1 *in vivo* and *in vitro*

Acyl-biotin exchange (ABE) assays followed by immunoprecipitation experiments showed that Drp1 *S-*palmitoylation normalized to total Drp1 was significantly decreased in cerebella from HOM mice (by >90% of WT or HET; Fig. 6e), confirming Drp1 as a key substrate of Zdhhc13. Further experimental confirmation of the *S*-palmitoylation activity of Zdhhc13 towards Drp1 was provided by showing their physical interaction in both *in vitro* and *in vivo* experiments. *In vitro* assays included the use of GST-tagged WT or L203X Zdhhc13 proteins immunocaptured by a high affinity anti-GST antibody pre-immobilized on a ForteBio Octet RED384 System biosensor and using recombinant, purified Drp1 (56, 167 and 500 nM; Fig. 6f). This analysis resulted in *k*_association_, *k*_dissociation_ and K_D_ of respectively 8.2 × 10^4^ M^−1^x s^−1^, 4.6 × 10^−2^ s^−1^ and 5.6 × 10^−7^ M for the WT and 2.1 × 10^4^ M^−1^x s^−1^, 1.8 × 10^−3^ s^−1^ and 8.8 × 10^−8^ M for the L203X Zdhhc13. These results indicated that Drp1 interacted strongly with both the WT and truncated forms of Zdhhc13, with the latter having a 6-fold higher affinity for Drp1 than the WT, but with no palmitoylating activity.

To avoid putative confounding effects of the GST tag, immunoprecipitation experiments utilizing whole brain lysates from WT and HOM mice were performed. Decreased levels of Drp1 were observed in HOM brain lysates vs. WT following IP with either anti-Drp1 or anti-Zdhhc13 antibodies (by 44% and 51% relative to WT; Fig. 6g and Supplementary Fig. S5). Notably, proteomics analysis revealed only 7 other co-precipitating proteins, from which, only one was a mitochondrial protein, the glutamate carrier 1 (GHC1 or SLC25A22; Supplementary Table S2 **)**.

Taken together, these results indicated that Zdhhc13 physically interacts with Drp1 (both *in vitro* and *in vivo*) and that both translocation and activity of Drp1 are strongly modulated by *S*-palmitoylation.

## Discussion

In a previous study, using a positional cloning approach, we have identified a nonsense mutation in exon 7 of *Zdhhc13* in *luc/luc* mice^3^. This T-A substitution, which results in a premature stop codon (L203X), generates a truncated form of the ubiquitously expressed Zdhhc13 protein representing a true loss-of-function mutation. Here, we show that Zdhhc13-mediated Drp1 *S*-palmitoylation, acting alone or in concert with others, is critical for sustaining mitochondrial dynamics and function and linked to neurobehavior. To our knowledge, this is the first report showing that loss of Drp1 *S*-palmitoylation by a specific PAT, Zdhhc13, disrupts brain mitochondria function and morphology eliciting abnormal behavior fueled by a shift towards glycolysis and excitatory-inhibitory neurotransmitter imbalances.

In agreement with a recent study^12^ conducted in liver of 6-w old Zdhhc13-deficient mice, Zdhhc13 loss-of-function leads to loss of palmitoylation of cytosolic and mitochondrial proteins in brain. While a database search of mitochondrial palmitoylated proteins resulted in >190 proteins in humans and mouse (~26 in rat) with an overlap of 121 (including Drp1; Dataset S1), the results presented in this study (i.e., direct protein-protein interaction between Zdhhc13 and Drp1 both *in vivo* and *in vitro*; decreased *S*-palmitoylation and mitochondrial co-localization in either *luc* mice or with pharmacological inhibition of *S*-palmitoylation activity; increased deficits in mitochondrial morphology, distribution and activity) are consistent with Drp1 as one of the substrates of Zdhhc13, and that *S*-palmitoylation of Drp1 (alone or in concert) affects its translocation to mitochondria likely contributing to the disrupted distribution and lower OXPHOS observed *in vivo*.

Lower OXPHOS was evidenced in HOM at 3-m (Complex IV activity) which progressed with age (Complex IV, V and citrate synthase), increased glycolysis and glutaminolysis (both HET and HOM), and enhanced perinuclear mitochondrial distribution (HeLa and NPC). The lower Complex IV activity from young mice (coupled with the decrease in Complex V in older mice) is consistent with other studies on Drp1 deficiency^40,41,48^. Considering the role of Drp1 in the modeling and organization of mitochondrial cristae^49^ and that disruption of mitochondrial fission is linked to impairment of mitochondrial ATP production^50^, a dysfunctional Drp1 would affect mitochondrial fission and mitophagy challenging the recycling of critical lipids for the mitochondrial inner membrane and the stabilization of respiratory chain super-complexes^51–53^. Naturally the relevance of Drp1 *S*-palmitoylation in the context of OXPHOS is enhanced in brain (vs. skin) because neurons are preferentially vulnerable to deficits in mitochondrial dynamics^39,54,55^. Indeed, the 20–30% cellular decrease in polarized mitochondria (*in vitro*), the 40–50% decrease in Complex IV activity (*in vivo* and *in vitro*), and the increased L/P ratio (*in vivo*) are consistent with the 22% decrease in ATP content in hippocampal mitochondria from conditional Drp1 KO mice^45^. While these deficits may be regarded as non-significant for the cellular energy balance, it is important to indicate that neurons rely heavily on mitochondria for localized energy support^56^. Loss of Drp1 causes depletion of mitochondria from synapses and dendrites^57^ leading to their structural loss^56^ and conditional KO mice develop progressive, neuronal subtype-specific alterations in mitochondrial shape and distribution in the absence of overt neurodegeneration with lower OXPHOS, ATP content, synaptic reserve pool vesicle recruitment and impaired spatial working memory^45^.

Synaptic dysfunction plays a critical role in the pathogenesis of Alzheimer’s disease^58^ and, as an extension, may elicit the behavioral changes observed in *luc* mice. The fact that Zdhhc13 deficiency had a major effect on cortical and less on cerebellar bioenergetics may be ascribed to differences in the neuron-to-glia ratio in cortex (1:4 ratio) vs. cerebellum [4:1^59^] and distinct rates of organelle turnover in these cell types. It is also possible that brain area-specific energy demands produce differential mitochondrial turnover rates^60,61^. Interestingly, differences in cortical vs. cerebellar Zdhhc13 requirement for mitochondrial function do not simply follow gene expression levels, as Zdhhc13 is expressed mainly in cerebellum more than cortex (Supplementary Fig. S3). These findings suggest that Zdhhc13-mediated Drp1 modifications may be also dependent on the presence of brain-specific interacting proteins required for mitophagy, fusion-fission processes, or protection of mitochondrial function^62^ with more overt mitochondrial deficits in those brain regions with critical roles in the circuitry of anxiety [cortex, amygdala, hippocampus, and striatum^32^], other than those tested here.

In regards to the neurochemistry underlying the behavioral deficits, the cerebellar metabolome was consistent with a switch towards aerobic glycolysis. While this switch will sustain rapid growth and proliferation at the expense of lower ATP production, this new steady-state may underlie the increases in glutamate and GABA (the major excitatory and inhibitory neurotransmitters, respectively, in the mature mammalian CNS, accounting for >90% of synapses^63^) observed in *luc* brains. Indeed, the glutamate-glutamine cycle accounts for a major fraction of glutamine synthesis^64^ with a metabolic rate similar to neuronal glucose oxidation^31,65^. Glutamate and GABA play major roles in energy metabolism, cortical excitability and cognitive function^65,66^. Alterations in Glu and GABA pathways could explain the behavioral deficits observed in *luc* mice as they are associated with many neurological and neuropsychiatric disorders such as Alzheimer’s, amyotrophic lateral sclerosis, mood disorders, high-functioning children with autism among others^67,68^. Although not tested in this study, it is conceivable that the co-precipitation of the mitochondrial glutamate carrier 1 by Drp1 and Zdhhc13 antibodies (Supplementary Table S2) may point to another target of this post-translational modification with critical relevance for neurotransmission (manuscript in preparation).

From a neurobehavioral standpoint, and considering the link between Drp1 and neurological diseases^44,69^, the modulation of mitochondrial fission-fusion in the context of *S*-palmitoylation by Zdhhc13 is of utmost relevance. A gene-dose-dependency was observed for the *luc* mice at 3-m of age in the increased sensory-gating activity, hypoactivity or hypolocomotion, decreased motor coordination, and decreased hindlimb grip strength, with no impact on learning and/or memory. Some of the subtle differences between HOM and HET in the behavioral and biochemical tests, which did not follow a gene-dose dependency, may be ascribed to compensatory processes (e.g., Fig. 2). In regards to behavior, our behavioral findings seem to represent a milder version of some of the aspects of HD^5^ (impaired rapid alternating movements and fine motor skills, dysarthria, ataxia and postural instability, gait and stance imbalance, broad-based gait and stance) but not others (cognition and memory^19,70^). The fact that the behavioral deficits in *luc* mice are not a phenocopy of HD’s features, provides additional evidence that Zdhhc13 has *other* roles than the palmitoylation of Htt (*vide infra;* also^71^), recalling some of the behavioral manifestations of other human diseases with an ataxia component (http://www.malacards.org/card/ataxia ^72^). Our *luc* mouse model (truncated Zdhhc13) shows a milder phenotypic version of that developed by Dr. Hayden’s KO (gene trapped^5^). This apparent discrepancy could be bridged by considering that these models were developed on different genetic backgrounds (C57BL/6NJ vs. FVB/N) as it has been observed in other cases (e.g., ref.^16^). The selection of the genetic background (C57BL/6NJ) of our *luca* model was based on the fact that the substrain C57BL/6 J has a negligible mitochondrial nicotinamide transhydrogenase activity due to a deletion comprising the *Nnt* gene (Supplementary Fig. S6), and as such, an increased background of mitochondrial oxidative stress^73^, which could interfere with the interpretation of our results on bioenergetics, likely leading to the presentation of a more severe phenotype. Indeed, the Zdhhc13-deficient mice on a mixed background^12^ showed poor weight gain despite an increased food consumption (factor that may relate to mitochondrial uncoupling or dysfunction) whereas no such differences were observed in the *luc* model.

**Figure 7:**
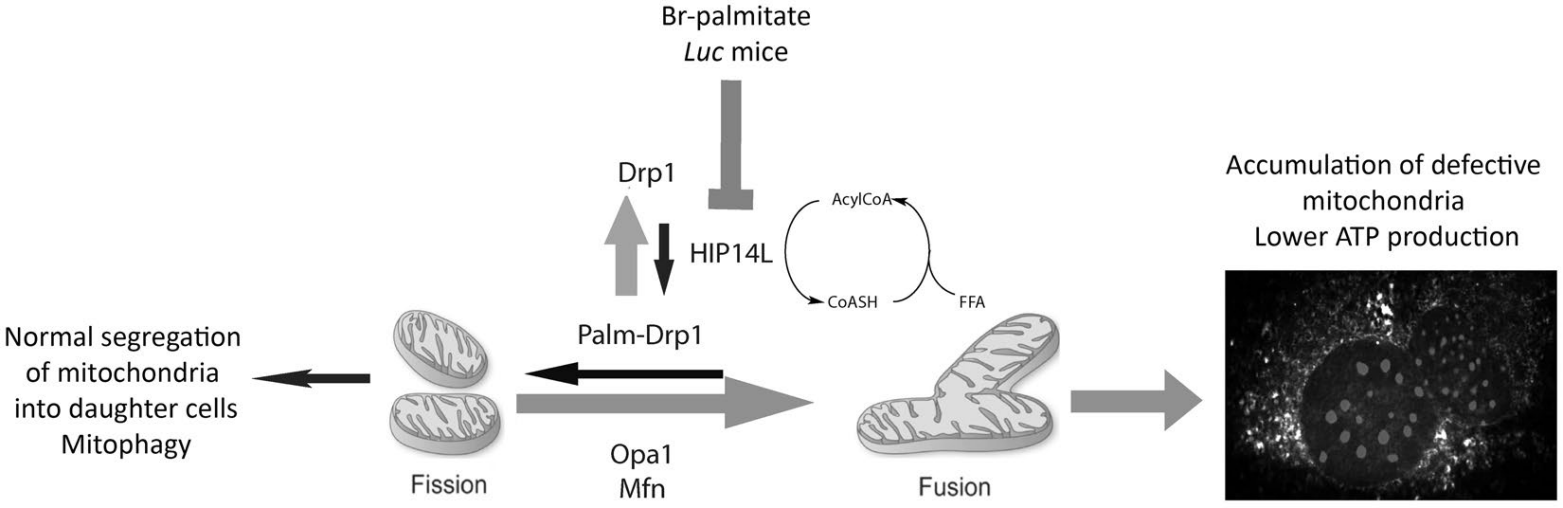
Graphical abstract of the role of Zdhhc13 in mitochondria dynamics. In *luc* mice or through pharmacological inhibition of *S*-palmitoylation, lower *S*-palmitoylation of Drp1 (Palm-Drp1) leads to impaired translocation to mitochondria. This results in an imbalance between the fusion and fission processes, interfering with mitophagy and/or segregation of mitochondria into daughter cells. Accumulation of defective mitochondria results in lower ATP production, and in the case of neurons, imbalances in neurotransmission. Both processes are likely to result in behavioral deficits observed in the *luc* mice.

In summary, for the first time this study shows evidence that increased Drp1 *S*-palmitoylation is a crucial mechanism for the translocation of Drp1 to mitochondria and the normal occurrence of the fission-fusion process. Abnormalities in this mechanism result in disrupted mitochondria morphology and distribution, which may affect not only cellular energy status but also neurotransmission and the integrity of synaptic structures in the brain underlying the behavioral abnormalities described in the *luc* mice. Considering the role of *S*-palmitoylated proteins in diverse processes, including neural development^74^, pancreatic beta cell metabolism^75^, and intermediary metabolism (this study and^12^), among others, we propose Drp1 *S*-palmitoylation as a novel, active process that enables the switch between mitochondria-derived ATP and glycolysis (including Glu and GABA cycles) by controlling mitochondria dynamics (Fig. 7) and, ultimately, affecting animal behavior (i.e., motor and non-motor, emotional skills). A metabolic deficit that leads to impaired ATP production and neurotransmitter pathways—as observed in this study—may explain the behavioral deficits exhibited by the *luc* model. Although we cannot completely exclude the role of other *S*-palmitoylated proteins (e.g., mitochondrial glutamate carrier 1) as well as other brain regions not tested in this study, *S*-palmitoylation of Drp1, acting alone or in concert (Supplementary Table S2), constitutes a novel regulation that has the potential to serve as a springboard to develop strategies for generating therapies not only for neurological disorders with energy deficits and/or neurotransmitter imbalances.

## Methods

### Origin of the *luc* mutation and phenotyping

The *luc* mutant allele (*Zdhhc13* ^*luc*^) was previously described^3^. The *Zdhhc13* ^*luc*^ allele was introgressed onto C57BL/6NJ by marker-assisted backcrossing to produce a full-congenic strain (C57BL/6NJ.Cg-*Zdhhc13* ^*luc*^, N10). Genotyping for the *Zdhhc13* ^*luc*^ allele was done by direct sequencing using PCR primers designed to detect the missense mutation on genomic DNA or by allele-specific KASPar PCR assay (Kbioscience, Beverly, MA). Additionally, all mice were also genotyped for the presence of the *Nnt* gene as described under the Supplementary Fig. S6.

### Animal housing

All mice were originally housed at MD Anderson Cancer Center Smithville followed NIH guidelines (Guide for the Care and Use of Laboratory Animals, 8^th^ Edition, 2010) in a facility accredited by the Association for Assessment and Accreditation of Laboratory Animal Care International. Male mice (3-month old) were then transferred to UC Davis and housed in the behavioral laboratory facility (Mouse Biology Program, UC Davis). At the beginning of experiments, mice were housed, 3–4 per cage separated by genotype in an animal room with constant temperature (22 ± 1 °C) and 12 h light/dark cycle (lights on/off at 6:00 a.m/p.m.), with free access to food and water. All experiments were performed in the early phase of the light cycle under standard room fluorescent lights. All methods and protocols were carried out in accordance with relevant NIH guidelines and regulations and approved by the IACUC Committee at the University of California Davis. Other details on animal general health and housing are reported in the Supplementary Methods and in Supplementary Fig. S1.

### Behavioral tests

Behavioral testing began 4–6 days after arrival into the animal facility when mice were still 3-months old. The sequence of experiments was designed from less to more intrusive in order to minimize the effects of previous experiments on future behaviors. Mice had at least 3 days separating between consecutive tests. Approximately, a week after the end of all behavioral testing and when mice were 4-m old, they were euthanized for biochemical analysis. The tests conducted in this study have been summarized below and detailed information has been included in the Supplementary Methods.

#### Open field and spontaneous locomotor activity

Spontaneous motor activity and anxiety-related behavior were recorded with well-established methods^17,19,20^. In parallel, routine parameters of general motor behaviors (e.g., rearing, speed, number and length of individual movements) were measured, providing accurate quantification of general locomotor activity^22^.

#### Acoustic startle

The acoustic startle response is routinely used to assess anxiety levels and sensor-motor gating^20^.

#### Grip strength

The grip strength was employed to assess the neuromuscular function as maximal muscle strength of forelimbs and combined forelimbs and hind limbs^21^.

#### Treadscan (treadmill)

The use of a treadmill to gather data for comprehensive, quantitative assessment of gait in mice is a sensitive method to evaluate motor performance. The computer-assisted footprint analysis assessed gait characteristics such as velocity, stance time, swing time, and stride length as the animal traverses a clear stationary walkway^76^.

#### Rotarod

This analysis was performed with the use of a Rotamex-5 4 Lane Rota-Rod for Mice with Photocell Detection (Columbus Instruments, Columbus, OH) in the dark phase and it was used to measure the latency of mice to fall from the rotating rod. One-training and three test sessions were performed the first day and then three test sessions per day for the next two days. For the training session animals were loaded onto rotating rod and tested for 120 s at 4 rpm. After 120 s, mice were removed, place in home cage for 60 s and test session started. Animals were loaded on the rotating rod and allowed to grasp the cylinder and begin a running motion. During each session, rod accelerated 1 rpm/10 s to a max of 40 rpm. If mice “clung” to the axle, and to prevent bias these were removed after three consecutive rotations and the latency at that time was used as the score for that animal. Tray and rod were cleaned with 10% Nolvolsan between groups of 4 and between days.

#### Passive avoidance

The passive avoidance test was performed as described^70^ to evaluate the learning capability of the three groups of mice.

### Protein *S*-palmitoylation quantification

Assessment of palmitoylation levels were carried out in cerebella from 4-m old mice and NPC samples as previously described by utilizing the acyl-biotin exchange assay^3^. More details are reported in the Supplementary Methods.

### Isolation of mitochondria from mouse brains

Enriched mitochondrial fractions were obtained from cortex and cerebellum of 4-month old and 2-y mice by mechanical cell disruption using a glass-Teflon homogenizer and subsequent centrifugation as previously described^77^. The enrichment of mitochondria in the mitochondria fraction was in the range of 30 to ~300-fold as judged by the ratios of mitochondrial over cytosolic markers (Supplementary Table S1). The mitochondria-enriched fraction was resuspended in hypo-osmotic 20 mM HEPES buffer, pH 7.4, supplemented with phosphatase inhibitors (Sigma #P0044) and proteolytic inhibitors (Sigma #P2714), and stored at −80 °C. A fraction of the mitochondria-enriched samples were further centrifuged at 16,000 *g* for 10 min and pellet (mitochondria membrane fractions) was resuspended in RIPA buffer for the quantification of membrane-bound palmitoyl-Drp1 levels (see below). Protein concentration was evaluated using a BCA Protein assay kit (Pierce) following manufacturer’s recommendation. Absorbance was recorded at 560 nm with the use of a Tecan Infinite M200 plate reader (Tecan, Austria). Other details were included in Supplementary Methods.

### Mitochondrial outcomes in NPC and cerebellum and cortex from *luc* mice

Protein extracts for enzymatic analyses were obtained by resuspending brain mitochondria (2–5 µg) or NPC in a hypotonic buffer (20 mM HEPES, pH 7.4) from 4-m and 2-y old mice and homogenizing the suspension with 30 strokes with a hand homogenizer, followed by two cycles of freezing and thawing. NADH-decylubiquinone oxidoreductase (Complex I), cytochrome *c* oxidase (Complex IV), ATPase (Complex V), and citrate synthase activities were evaluated as described in detail elsewhere^77,78^. Absorbance changes were followed in a Tecan M200 microplate reader and analyzed with the Magellan software, version 6. Changes in mtDNA CN were evaluated by dual-labeled qPCR. The gene copy number of ND1 was normalized by a single-copy nuclear gene (pyruvate kinase) as explained in detail before^78^.

### Cell culture conditions

Neuronal progenitor cells (NPC) were obtained from the CHDI repository program and grown as previously described^79^. Other details are reported in the Supplementary Methods. HeLa cells were cultured in Dulbecco’s modified Eagle’s medium (DMEM) with 10% fetal bovine serum (FBS), 1% nonessential amino acids (NEAA) and 100 U/ml penicillin-streptomycin and incubated at 37 °C with 5% CO_2_.

### Evaluation of mitochondrial distribution and morphology

NPC (1 × 10^5^) were seeded on sterile coverslips, grown overnight at 33 °C, followed by incubation with 0.5 µM MitoTracker Red CMXRos (MolecularProbes Inc., Eugene, OR) diluted in growth media for 30 min at 33 °C as previously described^79^. HeLa cells were transfected with GFP-tagged WT and truncated form of Zdhhc13 (L203X). The coding sequences of both the WT and L203X mutant *Zdhhc13* genes were unidirectionally cloned into the pEGFP-C1 vector (BD Biosciences, San Jose, CA) with the EGFP located in the *N*-terminal portion of the proteins. Transfection was confirmed by regular fluorescence microscopy. Seventy-two h after transfection cells were incubated with 0.5 µM MitoTracker Red CMXRos for 30 min at 37 °C and then washed three times with growth medium. Subsequently, cells were fixed with methanol for 20 min at −20 °C and later permeabilized with 5% newborn calf serum and 0.3% Triton X-100 in PBS for 30 min. Cells were then counterstained with DAPI for imaging with a ZeissObserverZ1 confocal microscope (CarlZeissMicroImagingInc., Thornwood,NY) or with an Olympus FV1000 laser scanning confocal microscope. Other details are reported in the Supplementary Methods.

### Zdhhc13-Drp1 protein-protein interaction

Kinetic parameters for the interaction of Drp1 with WT and truncated form of Zdhhc13 (L203X) were obtained with the use of an Octet RED384 system (Pall ForteBio, Menlo Park, CA). Briefly, purified recombinant WT (culture supernatant) and truncated Zdhhc13 (culture eluate) proteins were both produced by using the mouse Zdhhc13 sequence (UniProtKB: Q9CWU2). A GST-tagged recombinant, purified Drp1 (Novus Biologicals, # H00010059-P01) was immobilized onto a ForteBio biosensor, equipped with a high affinity pre-immobilized anti-GST antibody. Upon binding of the recombinant GST-tagged Drp1, the biosensor was exposed to either recombinant WT or mutant Zdhhc13 (bait proteins). The association and dissociation of bait proteins was monitored in parallel.

*In vivo* co-immunoprecipitation experiments were performed in total brains from WT and HOM mice homogenized in RPMI added with proteases and phosphatases inhibitors. One mg of total lysate was incubated with either an anti-Drp1 antibody (Cell Signaling; 1:100 dilution) or an anti-Zdhhc13 antibody (#4733, custom-made by Pacific Immunology, Ramona, CA; 1:50 dilution) overnight at 4 °C under gentle rocking. Subsequently, magnetic beads obtained from 50 µl of a Dynabeads suspension (Invitrogen) were added to the lysate-ab complexes and incubated at 20–22 °C for 30 min. Tubes were then placed on a magnet and supernatant discarded. Dynabeads-Ab-Ag complexes were then washed 3 times with PBS and finally added in loading buffer containing 50 mM dithiothreitol and heated at 70 °C for 10 m. Tubes were again placed on a magnet and supernatant collected and loaded in a 4–12% Bis-Tris gel and ran as previously described^3,79^. Proteins were then transferred onto a nitrocellulose membrane with the use of the iBlot system, blocked for 1 h at 20–22 °C with Li-COR blocking buffer and then incubated with the primary anti-Drp1 antibody (1:1000 dilution) overnight at 4 °C. Membranes were then washed 4 x for 5 min with TBST and then incubated with IRDye 800CW goat anti-rabbit antibody (LI-COR), diluted 1:10,000 for 1 h at 20–22 °C. Proteins were finally visualized with an Odyssey infrared imaging system LI-COR and densitometry performed with ImageJ. The antibody heavy chain bands at 55 kD were used as loading controls. In paralle, immunoprecipitation of proteins by using antibodies directed to Drp1 or Zdhcc13 was performed with whole brains from WT and HOM mice. The resulting pellets were subjected to mass spectrometry. All other details are under Supplementary Table S2.

### Statistical analysis

Statistical analysis of the data across the 3 genotypes was performed by ANOVA, followed by Bonferroni’s post-hoc test. To evaluate the contribution of time, genotype and brain area on the observed differences in mitochondrial outcomes a 2-way ANOVA was performed, followed by Bonferroni’s post-hoc test for individual comparisons. In all other instances an unpaired, two-tailed Student’s *t* test was employed.

## Electronic supplementary material

Supplementary Information

Dataset S1
