# Supplementary material for: Zdhhc13-dependent Drp1 S-palmitoylation impacts brain bioenergetics, anxiety, coordination and motor skills
Source: Sci Rep. 2017 Oct 16;7:12796. doi: 10.1038/s41598-017-12889-0 (PMC5643561; doi:10.1038/s41598-017-12889-0)
Supplement: Supplementary file 1 — Supplementary Information [file 41598_2017_12889_MOESM1_ESM.pdf]

Supplementary Information

Zdhhc13-dependent Drp1 *S*-palmitoylation impacts brain bioenergetics, anxiety, coordination and motor skills

Eleonora Napoli, Gyu Song, Siming Liu, Alexsandra Espejo, Carlos J. Perez, Fernando Benavides and Cecilia Giulivi

Supplementary Results

Lower mitochondrial mass, perinuclear distribution and condensed network in L203X Zdhhc13-overexpressing HeLa cells

Detailed morphology of mitochondria in primary neurons is quite difficult to resolve and we deemed of importance to test the deficit of Zdhhc13 in an independent biological model (HeLa cells). To this end, we overexpressed the GFP-labeled full-length and truncated form of Zdhhc13 (L203X) in an easy-to-transfect cell line, HeLa cells[1], to evaluate mitochondria content, function, shape and distribution (**Supplementary Figure S4**). Transfected cells were visualized by using the GFP fluorescent tag, and functional mitochondria by MitoTracker Red, which specifically stains polarized mitochondria. The full-length GFP-Zdhhc13 protein co-localized with a structure consistent with the Golgi apparatus, whereas the truncated form was characterized by a decreased staining and uniform cytoplasmic distribution (**Supplementary Figure S4a**). Polarized mitochondria, as well as the overall mitochondrial area, were decreased respectively by 20-30% and 50-70% in cells overexpressing the truncated Zdhhc13, accompanied by an increased clustering in the perikarya compared to the more evenly distributed mitochondrial network in cells treated with empty vector or overexpressing WT Zdhhc13 (**Supplementary Fig. S4a**). However, when the fluorescence intensity of polarized-functional mitochondria was normalized *per* mitochondrial area, a significant increase was obtained relative to WT Zdhhc13-transfected HeLa (by 1.8-fold, $p = 0.0023$). These results seemed consistent with a more compact mitochondrial distribution, possibly due to the presence of a hyperfused mitochondrial network. L203X Zdhhc13-overexpressing HeLa cells relative to WT Zdhhc13-overexpressing cells had lower polarized mitochondrial mass (by 21%; **Supplementary Figure S4b-c**), lower Drp1 in mitochondrial foci (by 23% normalized to polarized mitochondria; $p = 0.044$; **Supplementary Figure S4c**), whereas the amount of total (cellular) Drp1 was not significantly different between these two groups (**Supplementary Figure S4c**).

Supplementary Methods

Animals

The Institutional Animal Care and Use Committee reviewed all projects involving the use of experimental animals and conducts site inspections of our facilities every six months. Mice (3-month old) were then transferred to UC Davis and housed in the behavioral laboratory facility (Mouse Biology Program, UC Davis). Mice were first evaluated for general health [2,3], including

body weight, body length, eye, state of whiskers and (**Supplementary Fig. S1**). The animals (all males) were housed three to four mice/cage and provided with Purina 5058 chow and water *ad libitum*. The housing room was maintained at 23°C on a 12-h light/dark cycle (lights off at 6 pm). Mice from the three genetic groups appeared in good general health (checked by a third-party veterinary staff), without any overt impairments, aberrant responses or unusual levels of activity or fighting during the home cage observation periods. Eight out of nine homozygous and none of eight heterozygous mutant mice showed hair loss in different body areas (**Supplementary Fig. S1**). General health observations were performed upon arrival of mice at the behavioral laboratory facility. Behavioral testing began 4-6 days after arrival into the animal facility. Mice were characterized in assays for the presence of motor deficits (open field, rotarod, treadscan, and grip strength), anxiety (open field), and memory (passive avoidance test). Unless otherwise indicated, testing was conducted under fluorescent laboratory lighting. All mice appeared to be healthy at the conclusion of the testing sequence. At this point, when mice were 4-m old, they were euthanized for biochemical analysis.

Behavioral tests

Open field and spontaneous locomotor activity- Spontaneous motor activity and anxiety-related behavior were recorded with a well-established method [4-6]. Over a period of 10 min by measuring horizontal and vertical interruption of infrared PhotoBeams in a 16 inch × 16 inch arena of the SmartFrame Open Field System (Kinder Scientific) using MotorMonitor software. The open field test was conducted in the dark phase. The animal was placed into the center of the box and its behavior was monitored remotely by a videocamera. Measurements were taken of total activity, time spent in certain regions of the grid, number of vertical moves, and stereotypy counts. Animals were monitored for 20-min and then replaced into their home cage. After each animal was tested, the box was cleaned with 10% Nolvasan. This test provides indications of the presence of anxiety or anxiety-like behaviors, as inferred by the center and margin area and time occupied by the animals in the arena. In parallel, routine parameters of general motor behaviors (e.g., rearing, speed, number and length of individual movements) were measured, providing accurate quantification of general locomotor activity [7].

Grip strength- The grip strength was employed to assess the neuromuscular function as maximal muscle strength of forelimbs and combined forelimbs and hind limbs [8]. Forelimb and hindlimb grip strengths were measured as grasping applied by the mouse on a grid connected to a

sensor. Three trials were carried out in succession measuring forelimb-strength only, followed by three successive trials measuring the combined forelimb/hind limb grip strength. The apparatus consisted of a Force gauge meter, with included wire bar grip attachment (model E-DFE-002, Chatillon, Largo, FL) and PC equipped with the Animal Grip Strength System software (model 6910-0002-L, San Diego Instruments, San Diego, CA). For strength measurements, each mouse was lowered over the grid keeping the torso horizontal and allowing only its forepaws to attach to the top of grid before any measurements were taken. Its tail, ensuring the torso to remain horizontal, then gently pulled the mouse back and the maximal grip strength value was recorded. Three trials were performed for each mouse with a 1-min resting period between trials. In between mice, the wire bar grip was cleaned with 10% Nolvasan.

Treadscan (treadmill)- The use of a treadmill to gather data for comprehensive, quantitative assessment of gait in mice is a sensitive method to evaluate motor performance. The computer-assisted footprint analysis assessed gait characteristics such as velocity, stance time, swing time, and stride length as the animal traverses a clear stationary walkway[9,10]. The TreadScan software identified each individual paw of the mouse in each frame as it walked on the treadmill by measuring initial foot contact, stance duration, stride duration, foot liftoff, swing duration, stride length, track width, and toe-spread data for each foot. Gait analysis was evaluated measuring the following outcomes: stance time (paw on the belt), swing time (paw in the air), total stride time, stride length, foot contact area size (also representative of foot pressure), body-foot spacing distance (distances between body and feet), foot spacing distances (distance between various feet), running speed, stride frequencies, foot coupling. The apparatus used consisted of an automated treadmill (Clever Systems, Inc., Reston, VA), a Basler high-speed video camera and PC equipped with BCAM Cap Version 1.00. Each animal was placed into the treadmill enclosure and acclimated for 30 s. The treadmill was then turned on slowly to 20 cm/s and velocity increased to the highest speed tolerated by the animal. Video footage was analyzed using the Treadscan software.

Passive avoidance- The testing apparatus (consisting of Med Associates two chamber box, partition door, shock generator attached to dark chamber, false floor to cover shock grid in light chamber, light centered over light chamber to provide 800 lux brightness, and light shield for dark chamber) was a trough-shaped alley with two distinct compartments separated by a sliding door [11,12]. For the training trial, each mouse was randomly removed from home cage and

transferred immediately by tail-hold to the bright chamber. Measurements started as soon as the mouse touched the surface and was released. Ten s after release, the door was opened in the partition and the time taken to enter the second, darkened compartment was recorded. When the animal stepped into the dark section with all four paws, the door was closed and a 2-s footshock was given at 0.5 mA. After 10 s, the cover to the dark chamber was lifted and the mouse was removed and placed in its home cage. The chamber walls floor and shock grid were then cleaned thoroughly with 10% Nolvasan, the partition door removed and the dark chamber cover replaced. The training phase was repeated the following day. On the third day (time of the testing trial), the animal was returned to the white compartment and the latency to enter the dark compartment (to a maximum of 300 s) was recorded, but no shock was given. The procedure was repeated for all the mice tested.

Acoustic startle-The acoustic startle response was used to assess anxiety levels and sensor-motor gating [5]. At the start of each trial, the animal was given 5 min to acclimate to the testing apparatus. Mice were then placed on a platform device that records motion in an enclosed chamber allowing the mice to turn and freely move limbs and tail, but not to rear or ambulate. Five trial types, 10 trials each for a total of 50 trials per session were run. Trials were randomized within blocks of 5. The trial types were: (i) 120 dB startle alone, (ii) 120 dB startle with 74 dB prepulse, (iii) 120 dB startle with 82 dB prepulse, (iv) 120 dB startle with 90 dB prepulse, and (v) no stimulus –background white noise only. Apparatus frequency was broadband pink noise or white noise surrounding median of 5 khz. Each trial started with a 50 ms null period and ended with a 65 ms recording period. For both periods the background white noise was of 395 (70 dB).

Protein *S*-palmitoylation quantification

Tissues homogenates in RIPA (1 mg in protein) were pre-cleared of endogenous biotin-containing proteins by immunoprecipitation with streptavidin-coupled dynabeads (Invitrogen) following manufacturer's instructions. Upon removal of the magnetic beads, supernatants were then collected and treated overnight at 4°C with 20 mM *N*-ethylmaleimide (NEM) to block reduced, non-palmitoylated, cysteine residues. To remove the excess NEM, samples were subsequently precipitate with 10 % PCA for 10 min in ice and centrifugated at 13,000 x *g* for 10 min. Supernatant was carefully discarded and pellet was resuspended in 200 μl of chilled acetone, followed by centrifugation at 13,000 x *g* for 10 min. This wash step was repeated for a

total of 3 times, excess acetone was eliminated through speed vacuum centrifugation, and the final pellet was resuspended either in 1 M Tris, pH 7.4, or in 1 M hydroxylamine (HA), pH 7.4, for 1 h at room temperature, in order to cleave Cys-palmitoyl thioester bonds. After HA treatment, samples were passed 3 times through a Microcon with a cut-off of 3-kD centrifugal filter (Millipore, Billerica, MA) to eliminate the excess of HA. Elute was discarded and the concentrated samples were diluted to a final volume of 200 µl with PBS and incubated overnight at 4°C with the EZ-link BMCC-Biotin reagent (Pierce) to unveil total specific palmitoylation sites. Through one more round of washes through the Microcon filter units, samples were concentrated to a final volume of 10 µl, denatured in NuPAGE sample buffer and immunoblotting was performed as described in the "Western blotting" section. After blocking in Odyssey Blocking Buffer (LI-COR # 927-40000) for 1 h, membrane was incubated with the anti-biotin IRDye 680 Streptavidin antibody (LI-COR # 926-32231) for 1 h at RT. After 3 washes in TBST, palmitoylated proteins were visualized with an Odyssey infrared imaging system (LI-COR).

Isolation of mitochondria from cortex and cerebella of WT, HET and HOM mice

Enriched mitochondrial fractions were obtained from cortex and cerebellum of WT, HET and HOM mice by mechanical cell disruption using a glass-Teflon homogenizer and subsequent centrifugation as previously described [13]. Briefly, after asphyxiation of mice with CO_2, brains were quickly removed and cerebella and cortex were washed in cold 0.25 M sucrose, blotted and weighed. Then, they were placed in 0.22 M mannitol, 70 mM sucrose, 0.5 mM EGTA, 2 mM HEPES, 0.1% fatty acid-free BSA, pH 7.4 (MSHE), and homogenized in glass-Teflon homogenizer using a 5:1 buffer to brain wet weight ratio. Large cell debris and nuclei were pelleted by centrifuging at 600 × *g* for 5 min. Mitochondria were subsequently pelleted by centrifuging the supernatant for 10 min at 10,300 × *g*. This mitochondrial fraction was tested for enrichment and purity by performing proteomics of mitochondrial and post-mitochondrial fractions.

Cell culture conditions

Neuronal progenitor cells (NPC) were obtained from the CHDI repository program and grown as previously described in detail [14] . After thawing cells were plated in T75 flasks and grown at 33°C in a humidified atmosphere containing 5% CO_2 with 20 ml of Dulbecco's

Modified Eagle Medium supplemented with 10% FBS (Hyclone #SH30071.03) and 10^4 IU/ml penicillin and 10^4 μg/ml streptomycin (Gibco). When at 80–90% confluence, the cells were plated in T75 flasks at a cell density of 2.5×10^5 per flask and selected in DMEM with 400 μg/ml G418 (Invitrogen). Cells were grown at 33°C for 3–5 days, changing the media every 2 to 3 days, until 80–90% confluent and then harvested by trypsinization, counted with a TC-10 cell counter (Bio-Rad) with 0.1% Trypan Blue.

HeLa cells were cultured in Dulbecco's modified Eagle's medium (DMEM) with 10% fetal bovine serum (FBS), 1% nonessential amino acids (NEAA) and 100 U/ml penicillin-streptomycin and incubated at 37°C with 5% CO_2.

Evaluation of mitochondrial distribution and morphology by confocal microscopy in cells in culture

Striatal progenitor cells (1×10^5) were seeded on sterile coverslips, grown overnight at 33°C, followed by incubation with 0.5 μM MitoTracker Red CMXRos (MolecularProbes Inc., Eugene, OR) diluted in growth media for 30 min at 33°C. After staining, cells were washed with media and fixed in 3.7% formaldehyde for 10 min. Fixed cells were washed again in PBS, blocked/permeabilized for 30 min in blocking buffer at 20–22°C, incubated overnight with an anti-Drp1 antibody (1:500 dilution; Cell Signaling Technology, #8570S). Immunoreactivity was visualized using an Alexa-Fluor555-conjugated anti-rabbit IgG (Invitrogen #A32732) secondary antibody for 1 hour at room temperature. Coverslips were subsequently counterstained with 1 μg/ml 4',6'-diamidino-2-phenylindole (DAPI), and mounted on glass slides with Prolong antifading mounting media (Life Technologies).

HeLa cells were transfected with GFP-tagged WT and truncated form of Zdhhc13 (L203X). The coding sequences of both the WT and L203X mutant *Zdhhc13* genes were unidirectionally cloned into the pEGFP-C1 vector (BD Biosciences, San Jose, CA) with the EGFP located in the *N*-terminal portion of the proteins. Transfection was confirmed by regular fluorescence microscopy. Cells were fixed, blocked and stained with DAPI for imaging with a Zeiss Observer Z1 confocal microscope (Carl Zeiss MicroImaging Inc., Thornwood, NY). Seventy-two hours after transfection cells were incubated with 0.5 μM MitoTracker Red CMXRos for 30 min at 37°C and then washed three times with growth medium. Subsequently, cells were fixed with methanol for 20 min at -20°C and later permeabilized with 5% newborn calf serum (NCS) and

0.3% Triton X-100 in phosphate-buffered saline (PBS) for 30 min. To detect the expression of endogenous Drp1, the cells were incubated overnight with a 1:200 dilution (in 1% BSA and 0.3% Triton X-100 PBS) of rabbit anti-Drp1 (Cell Signaling Technology, #8570S). Immunoreactivity was visualized using an Alexa-Fluor555-conjugated anti-rabbit IgG (Invitrogen #A32732) secondary antibody for 1 h at room temperature. DNA was stained with DAPI. Fluorescent images were obtained using an Olympus FV1000 laser scanning confocal microscope with either a 40X or a 60X objective. Mitochondrial morphology was evaluated by ImageJ using the parameters described in detail before [14].

Supplementary Table S1: Enrichment and purity of mitochondrial fractions						
	Gene symbol	Abundance in cytosolic fraction	Cytosolic abundance normalized to Ldhb (a)/Ldhb	Abundance in mitochondrial fraction	Mitochondrial abundance normalized to Ldhb (b)/Ldhb	Enrichment in mitochondrial fraction
		(a)	(c)	(b)	(d)	(d/c)
**Plasma membrane**	Nt5c3a	0.3	0.003	0.000	0.000	0
	Atp1a1	11.9	0.133	149.1	2.473	18.5
	Atp1a2	3.6	0.040	103.3	1.713	42.6
	Atp1b1	5.6	0.063	44.7	0.742	11.8
	Atp1b2	1.1	0.013	51.3	0.851	66.2
**Cytosol**	Ldha	45.1	0.508	33.0	0.547	1.1
	Ldhb*	88.9	1.000	60.3	1.000	1.0
**Lysosomes**	Ppal	nd		nd		nd
**Peroxisomes**	Glo1	21.6	0.243	8.0	0.133	0.5
**Mitochondria**	Cs	0.3	0.003	52.0	0.862	268.3
	Cox5a	0.3	0.003	59.9	0.993	308.9
	Cox5b	0.1	0.002	62.9	1.042	648.7
	Glud1	0.9	0.010	46.0	0.763	79.1
	Atp5b	3.3	0.037	512.7	8.503	230.1
	Vdac1	2.6	0.029	52.1	0.865	29.9
**Microsomes**	Hspa5	30.4	0.342	103.3	1.713	5.0
	Pdia3	35.3	0.397	80.7	1.339	3.4
**Glia**	Gfap	0.7	0.008	2	0.033	4.1
**Neuron**	Stx1a	0.7	0.008	53.7	0.891	110.9
	Abpa1	1.4	0.016	1.6	0.026	1.6
	App	5.1	0.058	7	0.116	2.0
**Myelin**	Cnp	8.9	0.100	128.4	2.130	21.4
	Mbp	1.6	0.018	20.3	0.336	19.0
**Synaptosome**	Syn1	39.0	0.439	230.7	3.826	8.7
	Sv2a	8.4	0.095	38.7	0.642	6.8
	Sv2b	0.1	0.002	26.1	0.434	269.8

Estimates of the proportion of mitochondria recovered and the degree of purification achieved in each of the fractions named “cytosol” and “mitochondria (n = 7 mice) was obtained using proteomics analysis in each of these fractions. Data are shown as the average of 7 animals. In assessing the suitability of mitochondrial preparation for our studies, the degree of contamination by non-mitochondrial subfractions is of greater importance than estimates of the purification (enrichment) based on mitochondrial markers and activities. A specific marker for synaptosomes is usually Ldh. The presence of this protein in mitochondrial preparations provides a measure of

the amount of cytoplasmic material present in the mitochondrial fraction in the form of synaptosomes or other membrane-enclosed vesicles. Contamination with any of the subcellular compartments tested was low as indicated by the relative ratio of the compartment marker normalized by the cytosolic marker Ldh isoform b (indicated with asterisk). Similarly, the contamination with glia was minimal (Gfap = 4) as indicated by the recovery of Gfap, whereas altogether the markers of neurons, synaptosomes, and myelin (average 55), seemed more significant suggesting that the origin of the mitochondria was mainly neuronal. The enrichment of mitochondria in the mitochondria fraction was in the range of 30 to ~300. Abbreviations: nd, not detected.

Supplementary Table S2: List of common proteins pulled down by antibodies against either Drp1 or Zdhhc13

UniProt	Protein	Gene	Function
**P11798**	Calcium/calmodulin-dependent protein kinase type II subunit alpha	Camk2a	Prominent kinase in the central nervous system. Involved in long-term potentiation, synaptic plasticity and neurotransmitter release.
**Q8VDF3**	Death-associated protein kinase 2	Dapk2	Calcium/calmodulin-dependent serine/threonine kinase involved in cell survival, apoptosis, and autophagy.
**Q8K1M6**	**Dynamin-1-like protein**	**Dnm1l**	**Mediates mitochondrial membrane fission through a GTP hydrolysis-dependent mechanism. Required for normal brain development, including that of cerebellum.**
**P16858**	Glyceraldehyde-3-phosphate dehydrogenase	Gapdh	Key enzyme in glycolysis. Modulates the organization and assembly of the cytoskeleton. Also participates in transcription, RNA transport, DNA replication and apoptosis.
**P62827**	GTP-binding nuclear protein Ran	Ran	GTPase involved in nucleo-cytoplasmic transport of proteins and RNAs. Controls cargo loading and release by transport receptors in the proper compartment and ensure the directionality of the transport.
**Q80X52**	Mitochondrial glutamate carrier 1	Slc25a22	Involved in the transport of glutamate across the inner mitochondrial membrane.
**P61264**	Syntaxin-1B	Stx1b	Potentially involved in docking of synaptic vesicles at presynaptic active zones.
**Q99104**	Unconventional myosin-Va	Myo5a	Actin-based motor. Mediates the transport of vesicles to the plasma membrane. May also be required for some polarization process involved in dendrite formation.

Drp1 or Zdhhc13 were pulled down by IP of brain lysates from WT or KO mice. The resulting pellets under each condition were analyzed by mass spectrometry. Quantification was performed by spectral counting. Abundance of proteins pulled down in WT were subtracted from those pulled down in KO mice (used as background) for each antibody used. Once these two lists of proteins were obtained (one with each antibody), only common proteins were selected. Mass spectrometry was performed at the UCD Facility.

Legends to Supplementary Figures and Dataset

Supplementary Figure S1 Functional observation battery tested in mice utilized in this study

This battery consists of non-invasive procedures designed to test the physiological and functional status of mice upon receipt. The following processes are assessed via simple handling of the animal, which takes between 10-15 min *per* animal: (a) Neuromuscular and activity (gait, righting reflex, landing foot splay, rearing, posture, general activity = gait and tail hang); (b) Physiological (body weight and length, sex, eye status, signs of fighting, whiskers = length and position), and body temperature (not shown). Any other unusual appearance (such in this case, baldness) is reported also under the comments section.

Supplementary Figure S2 Body parameters and behavioral outcomes in *luc* mice

Body weight, length, grip strength and treadmill outcomes are reported as mean ± SEM. Number of animals tested were 8 WT, 8 HET and 9 for HOM, all of which were 3 m of age.
Grip strength: Grip strength test was performed to evaluate neuromuscular functions by determining the maximal peak force developed by fore and hind limbs in WT, HET and HOM mice. Statistical analysis was performed by ANOVA followed by Bonferroni's post-hoc test. $^{**}p = 0.0063$. *Treadmill:* Gait analysis was evaluated measuring outcomes shown in **Fig. 2**, as well as the following: speed (speed at which the animal ran, as rpm/min), average print area (average of the size of the foot print over the entire stance), foot stance base (distance between the midpoint of the trajectory of the front foot stance and the midpoint of the trajectory of the rear foot stance), average break (time elapsed between start of a stance and the instant the foot reaches the normal stance position of the feet), propel (time elapsed between the instance the foot reaches normal stance position and time when it leaves floor surface), and closest and furthest distance during the stance phase the foot attained relative to long and short body axes. No statistically significant differences in speed, foot stance base, and propel were observed among the three genotypes. FR = front right; FL = front left; RR = rear right; RL = rear left. Reported are outputs significantly different among the three groups, shown as mean ± SEM for fore and hind limbs of right and left side of the body. Statistical analysis was performed with a one-way ANOVA followed by Bonferroni's post-test for multiple comparisons. P values are as follows: *** ≤ 0.0001; * = 0.019, 0.021, 0.038 and 0.047 respectively for (i) FL average print area, (ii)

RR average break, (iii) furthest distance attained by FR relative to long body exes during the stance phase, and (iv) furthest distance attained by RL relative to long body exes during the stance phase.

Supplementary Figure S3   Human Zdhhc13 expression

Data obtained from http://www.proteinatlas.org/ENSG00000177054-ZDHHC13/tissue) [15].

Supplementary Figure S4   Mitochondria morphology/distribution and Drp1 levels in HeLa cells overexpressing GFP-tagged WT or L203X Zdhhc13

(**A**) GFP staining and mitochondrial distribution in HeLa cells transfected with EV, overexpressing (OE) WT Zdhhc13 or L203X truncated Zdhhc13. Green = GFP; Blue = DAPI. Mitochondria (in gray) show a more perinuclear distribution in L203X Zdhhc13-transfected HeLa cells, relative to non-transfected or WT Zdhhc13-overexpressing cells. In terms of mitochondrial morphology, L203X Zdhhc13-transfected cells show decreased functional mitochondrial mass, increased perinuclear and hyperfused morphology (evidenced by the higher higher MitoTracker staining *per* mitochondria area). Pictures were taken at a 180x magnification. Surface plots, showing cellular mitochondrial distribution and localization, were computed with the ImageJ software. **(B)** GFP staining and mitochondrial morphology and distribution in HeLa cells transfected with EV, overexpressing WT Zdhhc13 or L203X Zdhhc13. Green = GFP; Blue = DAPI. Mitochondria (in gray) show a more perinuclear distribution, as well as decreased functional (as decreased mitotracker fluorescence intensity *per* cell area) and total mitochondrial mass (as decreased mitochondrial area *per* cell) in L203X Zdhhc13-transfected HeLa cells, relative to non-transfected or WT Zdhhc13-overexpressing cells. Pictures were taken at a 60x magnification, zoom of 3. Densitometry and mitochondrial morphology was evaluated with the ImageJ software (see Methods). Data are shown as average ± SEM of ≥50 GFP-positive cells. Statistical analysis was performed by ANOVA, followed by Bonferroni's post-hoc test. P values are as follows: a = 0.041; b = 0.031; c = < 0.0001; d = 0.023; e = 0.002. FI = Fluorescence Intensity. (**C**) GFP, Mitotracker DeepRed and Drp1 staining was performed as described in the Methods. Pictures were taken with a 63x magnification, zoom of 1. Data are expressed as average ± SEM of ≥50 GFP-positive imaged cells for WT or L203X overexpressing cells. Mitochondrial Drp1 levels were evaluated using the ImageJ (Fiji) colocalization plugin and

normalized by functional mitochondrial mass. Statistical analysis was performed by two-tailed Student's t test.

Supplementary Figure S5 Full length immunoblot images of Drp1 and palmitoyl-Drp1 levels in WT, HET and HOM, and for the Drp1-Zdhhc13 protein interaction

(**a**) Drp1, VDAC and actin levels in cytosolic and mitochondrial cerebellar fractions from WT, HET and HOM mice. (**b**) Drp1, beta-subunit of mitochondrial ATPase and actin levels in total and mitochondrial fractions of NPC before and after treatment with 20 μM 2-bromo-palmitate for 6 h. * Unspecific band. (**c**) Cerebellar Drp1 palmitoylation levels. Original images obtained with the use of the Odyssey Licor imaging system as described in the Methods and Supplementary Methods. Green: Drp1 image obtained through the 800 channel laser at 785 nm; red: palmitoylation signal obtained through the 700 channel laser at 685 nm; yellow: overlay of red and green signal showing palmitoyl-Drp1. (**d**) Drp1 levels in WT and HOM brains upon immunoprecipitation with anti Drp1 or Zdhhc13 antibody. HC = heavy antibody chain; LC = light antibody chain.

Supplementary Figure S6 Representative *Nnt* genotyping of mice utilized in this study

All mice utilized in this study were genotyped for the wild-type or truncated *Nnt* as described [16,17]. Genomic DNA was extracted from tails using a Qiagen DNeasy tissue kit. Concentration and purity of DNA was measured at an absorbance of 260 nm and 280 nm on a Tecan Infinite M200 Nanoquant (Tecan, Austria). The *Nnt* allele expressed by the B6/NJ substrain was PCR genotyped using a three primer, two allele PCR assay that discriminates between the *Nnt* wild-type allele and the mutant allele lacking exons 7–11 inclusive found in B6/J mice. Primers were designed using the mouse genome sequence of the *Nnt* locus available from the public Ensembl website. The primer sequences are (all 5′–3′): *Nnt*-COM (GTAGGGCCAACTGTTTCTGCATGA); *Nnt*-WT (GGGCATAGGAAGCAAATACCAAGTTG); *Nnt*-MUT (GTGGAATTCCGCTGAGAGAACTCTT). The "COM" or common primer participates in amplification of both the wild type and NntB6J mutant alleles, while the "WT" and "MUT" primers are specific to the wild type and NntB6J mutant alleles, respectively. The amplification products are 579 bp for the wild-type allele and 743 bp for the mutant allele. Use of the primers with heterozygous mutant NntB6J template produced an additional faint product at 1 kb that assisted in genotype assignment. Amplification

conditions used were initial melt 95°C, 5 min; then 35 cycles of 95°C, 45 s, 58°C, 30 s, 72°C, 45 s; followed by a final extension of 5 min at 72°C. Products were analyzed by electrophoresis through a 1% agarose, 1× TBE gel followed by staining in ethidium bromide and visualized using a UV light box and a commercial imaging system (KodakImager 2000MM). Labels: *Nnt*$^{+/+}$, $^{+/-}$ and $^{-/-}$ are positive controls; the numbers indicate the mouse identification numbers; negative control (no DNA added)

Supplementary Dataset S1 Mitochondrial *S*-palmitoylated proteins in human, mouse and rat

Mitochondrial *S*-palmitoylated proteins in human, mouse and rat. Bolded and highlighted are subunits of Complex I, IV and V, citrate synthase, proteins involved in GABA metabolism, and Drp1. Mitochondrial palmitoylated proteins for each species were obtained through the SwissPalm database [18] as of June 7th, 2017.

References

1 Izsvak, Z., Chuah, M. K., Vandendriessche, T. & Ivics, Z. Efficient stable gene transfer into human cells by the Sleeping Beauty transposon vectors. *Methods* **49**, 287-297, doi:10.1016/j.ymeth.2009.07.001 (2009).

2 Crawley, J. N. Behavioral phenotyping of transgenic and knockout mice: experimental design and evaluation of general health, sensory functions, motor abilities, and specific behavioral tests. *Brain Res* **835**, 18-26 (1999).

3 Napoli, E. *et al.* Mitochondrial dysfunction in Pten haplo-insufficient mice with social deficits and repetitive behavior: interplay between Pten and p53. *PLoS One* **7**, e42504, doi:10.1371/journal.pone.0042504 (2012).

4 Breuer, M. E., Willems, P. H., Russel, F. G., Koopman, W. J. & Smeitink, J. A. Modeling mitochondrial dysfunctions in the brain: from mice to men. *J Inherit. Metab. Dis.* **35**, 193-210, doi:10.1007/s10545-011-9375-8 (2012).

5 Crawley, J. N. Behavioral phenotyping strategies for mutant mice. *Neuron* **57**, 809-818, doi:10.1016/j.neuron.2008.03.001 (2008).

6 Hickey, M. A. *et al.* Extensive early motor and non-motor behavioral deficits are followed by striatal neuronal loss in knock-in Huntington's disease mice. *Neuroscience* **157**, 280-295, doi:10.1016/j.neuroscience.2008.08.041 (2008).

7 Borlongan, C. V. *et al.* Hyperactivity and hypoactivity in a rat model of Huntington's disease: the systemic 3-nitropropionic acid model. *Brain Res Brain Res Protoc* **1**, 253-257 (1997).

8 Lone, A. M. *et al.* Deletion of PREPl causes growth impairment and hypotonia in mice. *PLoS One* **9**, e89160, doi:10.1371/journal.pone.0089160 (2014).

9 Hamers, F. P., Koopmans, G. C. & Joosten, E. A. CatWalk-assisted gait analysis in the assessment of spinal cord injury. *J Neurotrauma* **23**, 537-548, doi:10.1089/neu.2006.23.537 (2006).

10 Hamers, F. P., Lankhorst, A. J., van Laar, T. J., Veldhuis, W. B. & Gispen, W. H. Automated quantitative gait analysis during overground locomotion in the rat: its application to spinal cord contusion and transection injuries. *J Neurotrauma* **18**, 187-201, doi:10.1089/08977150150502613 (2001).

11 Cowin, R. M. *et al.* Onset and progression of behavioral and molecular phenotypes in a novel congenic R6/2 line exhibiting intergenerational CAG repeat stability. *PLoS One* **6**, e28409, doi:10.1371/journal.pone.0028409 (2011).

12 Schrott, L. M. & Crnic, L. S. Sensitivity to foot shock in autoimmune NZB x NZW F1 hybrid mice. *Physiol Behav* **56**, 849-853 (1994).

13 Giulivi, C. *et al.* Basal bioenergetic abnormalities in skeletal muscle from ryanodine receptor malignant hyperthermia-susceptible R163C knock-in mice. *J Biol Chem* **286**, 99-113, doi:10.1074/jbc.M110.153247 (2011).

14 Napoli, E. *et al.* Defective mitochondrial disulfide relay system, altered mitochondrial morphology and function in Huntington's disease. *Hum Mol. Genet.* **22**, 989-1004, doi:10.1093/hmg/dds503 (2013).

15 Uhlen, M. *et al.* Proteomics. Tissue-based map of the human proteome. *Science* **347**, 1260419, doi:10.1126/science.1260419 (2015).

16 Nicholson, A. *et al.* Diet-induced obesity in two C57BL/6 substrains with intact or mutant nicotinamide nucleotide transhydrogenase (Nnt) gene. *Obesity (Silver Spring)* **18**, 1902-1905, doi:10.1038/oby.2009.477 (2010).
17 Ronchi, J. A. *et al.* A spontaneous mutation in the nicotinamide nucleotide transhydrogenase gene of C57BL/6J mice results in mitochondrial redox abnormalities. *Free Radic Biol Med* **63**, 446-456, doi:10.1016/j.freeradbiomed.2013.05.049 (2013).
18 Blanc, M. *et al.* SwissPalm: Protein Palmitoylation database. *F1000Res* **4**, 261, doi:10.12688/f1000research.6464.1 (2015).

Genotype	Sex	Weight (g)	Length (mm)	Eyes	Signs of Fighting	Whiskers	Tail Hang	Gait	Comments
WT	M	26.34	99	Open	No	normal	0	1	
WT	M	25.32	97	Open	No	normal	0	1	
WT	M	25.28	97	Open	No	normal	0	1	
WT	M	27.31	100	Open	No	normal	0	1	
WT	M	28.58	99	Open	No	normal	0	1	
WT	M	24.91	98	Open	No	normal	0	1	
WT	M	25.59	92	Open	No	normal	0	1	
WT	M	21.51	85	Open	No	normal	0	1	Right eye smaller than left
HET	M	25.02	93	Open	No	normal	0	1	
HET	M	24.21	95	Open	No	normal	0	1	
HET	M	24.28	90	Open	No	normal	0	1	
HET	M	24.41	93	Open	No	normal	0	1	
HET	M	25.76	94	Open	No	normal	0	1	
HET	M	24.17	93	Open	No	normal	0	1	
HET	M	30.92	96	Open	No	normal	0	1	
HET	M	30.3	98	Open	No	normal	0	1	
HOM	M	24.39	91	Open	No	normal	0	1	Hair Loss (neck, eyes, base of tail, left flank)
HOM	M	25.64	87	Open	No	short	0	1	Hair Loss (back and face)
HOM	M	25.26	92	Open	No	normal	0	1	Hair Loss (neck, flanks, back, eyes)
HOM	M	24.88	88	Open	No	normal	0	1	Hair Loss (flanks, back, neck)
HOM	M	25.43	92	Open	No	normal	0	1	Hair Loss (rear flanks, abdomen, neck)
HOM	M	25.59	90	Open	No	normal	0	1	Hair Loss (both flanks, abdomen, eyes, neck)
HOM	M	23.84	88	Open	No	normal	0	1	Inflamed right eyelid
HOM	M	24.28	89	Open	No	normal	0	1	Hair Loss (back, flank, neck, eyes)
HOM	M	26.52	90	Open	No	normal	0	1	Hair Loss (entire back and abdomen)

COLUMN LABEL	DESCRIPTION
Tail Hang	0-limbs splayed outward
	P-hindlimbs drawn toward each without touching -or-one leg is drawn to the body
	F-both legs pulled, in tightly, either touching each other, or touching the body
Gait	Normal/Abnormal
1	Normal gait
2	Ataxia. Excessive sway, rocks or lurches
3	Hind limbs exaggerated or overcompensated, drag, or splayed
4	Feet markedly point outward from body
5	Forelimbs drag, are extended, or unable to support weight
6	Walks on tiptoes
7	Hunched or crouched body position
8	Body drags or is flattened against surface

Supplementary Figure S1

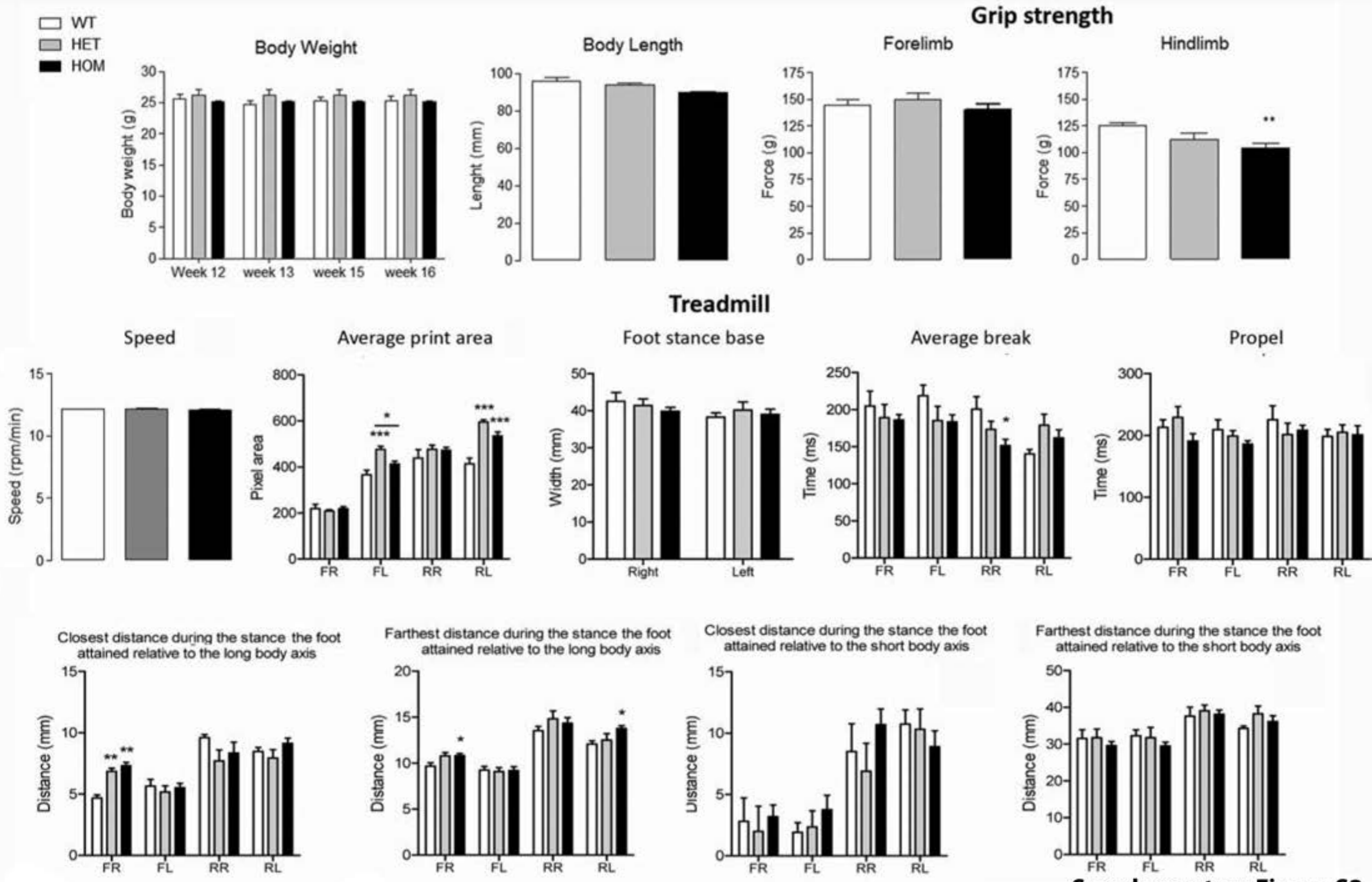

Supplementray Figure S2

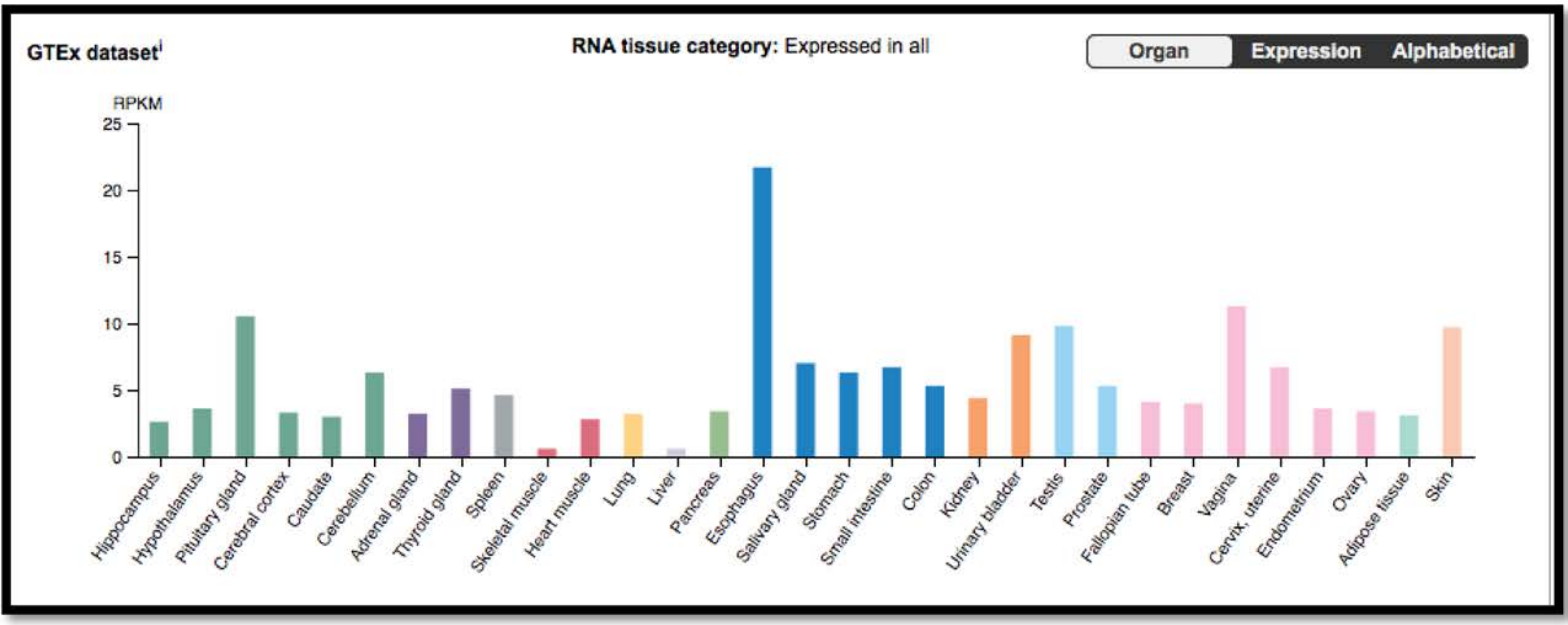

Supplementary Figure S3

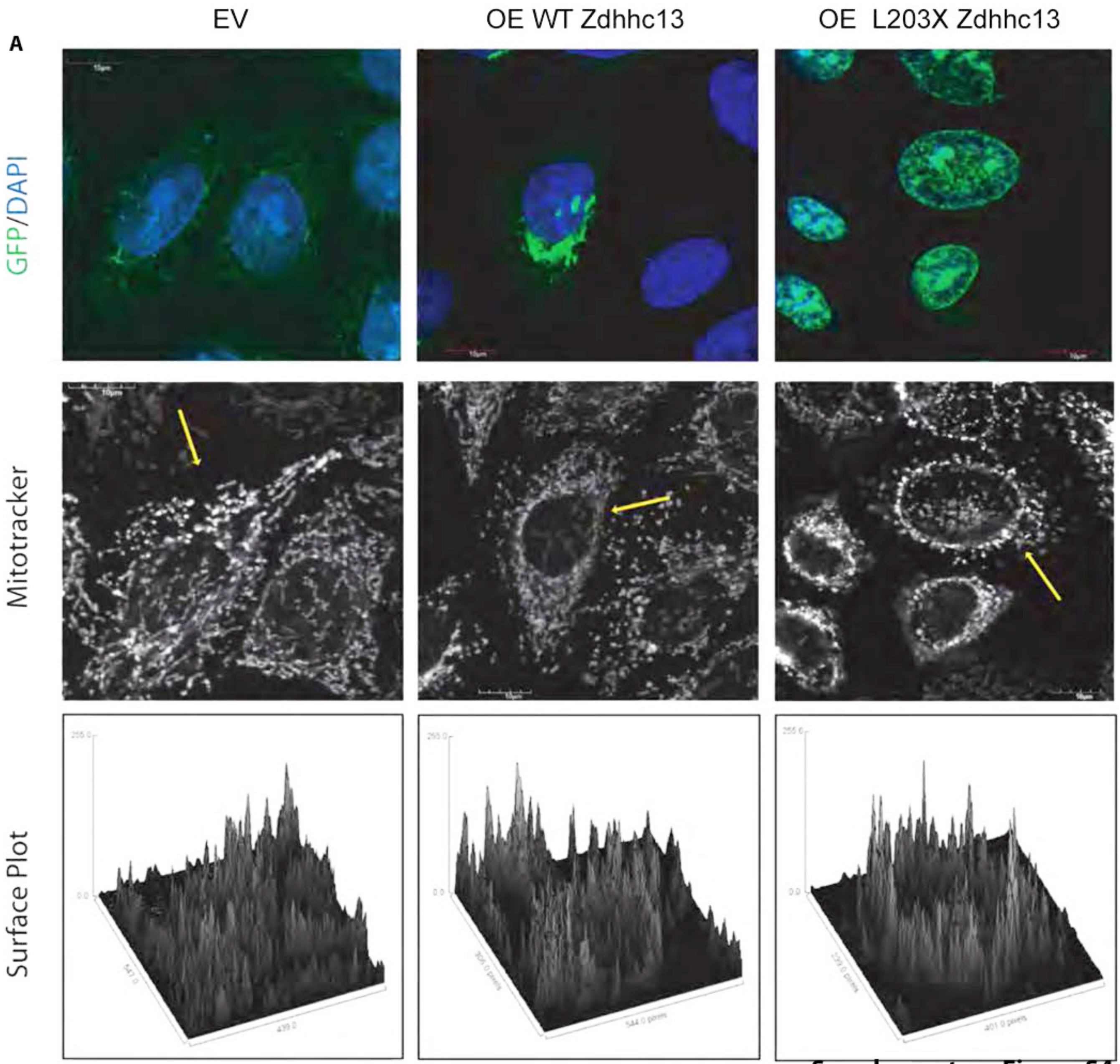

Supplementary Figure S4

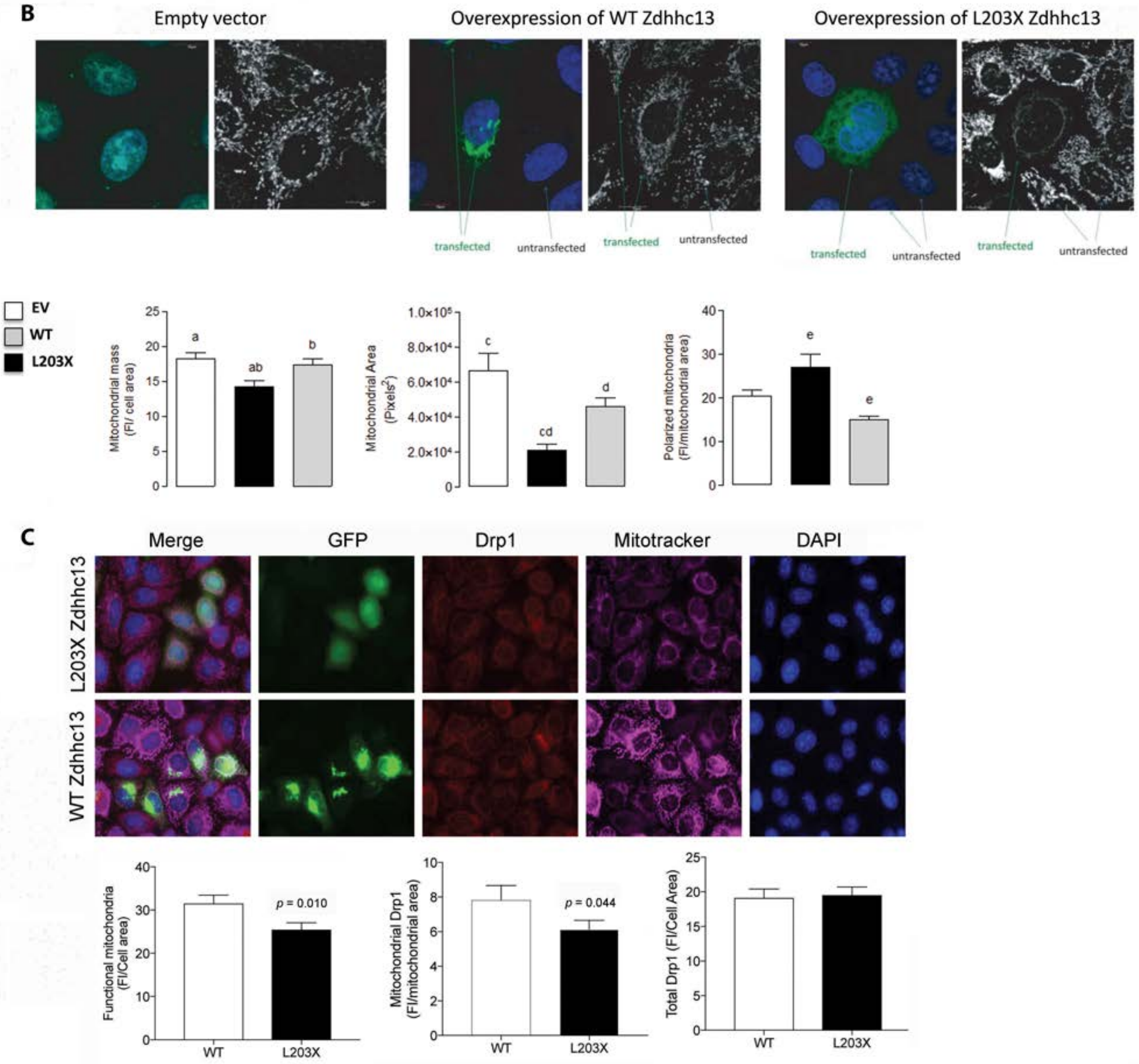

Supplementary Figure S4

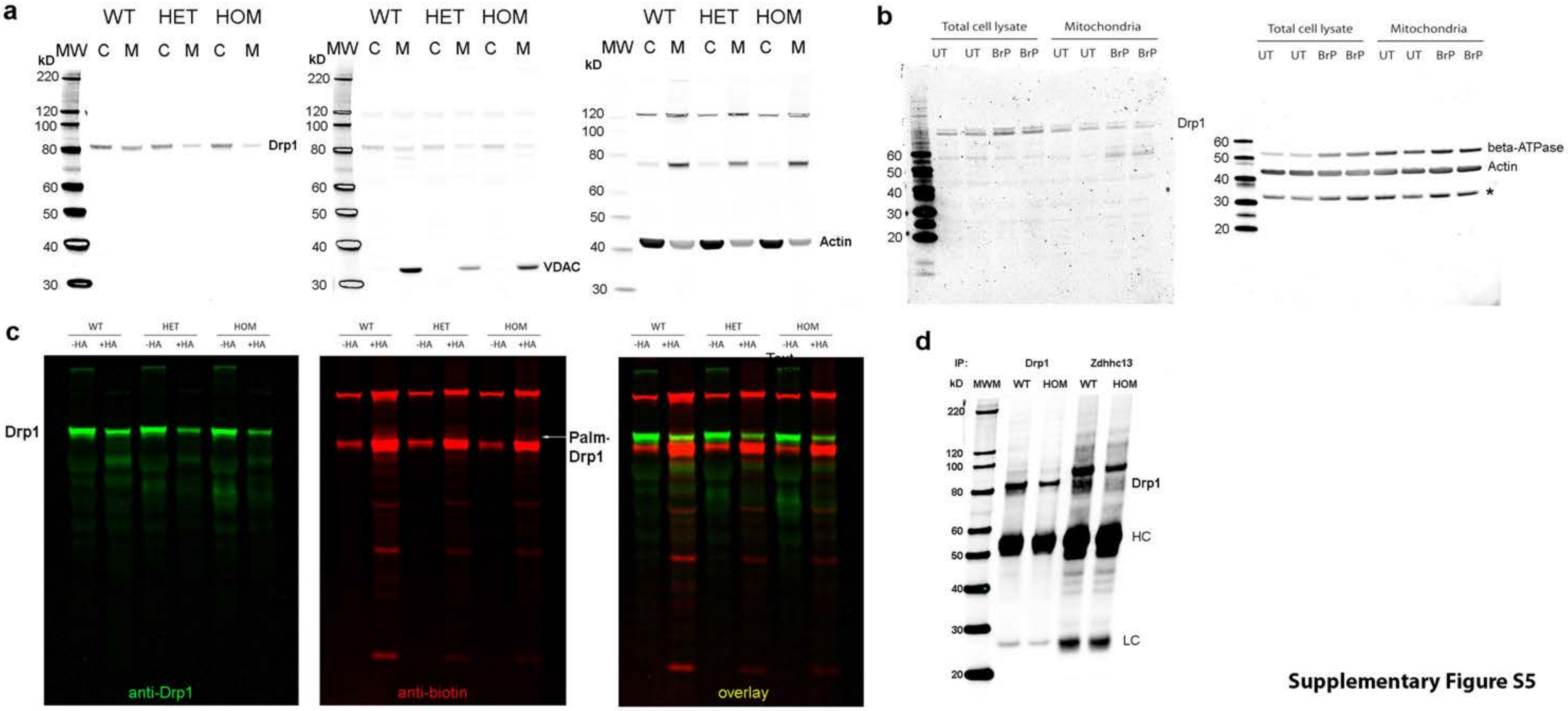

Supplementary Figure S5

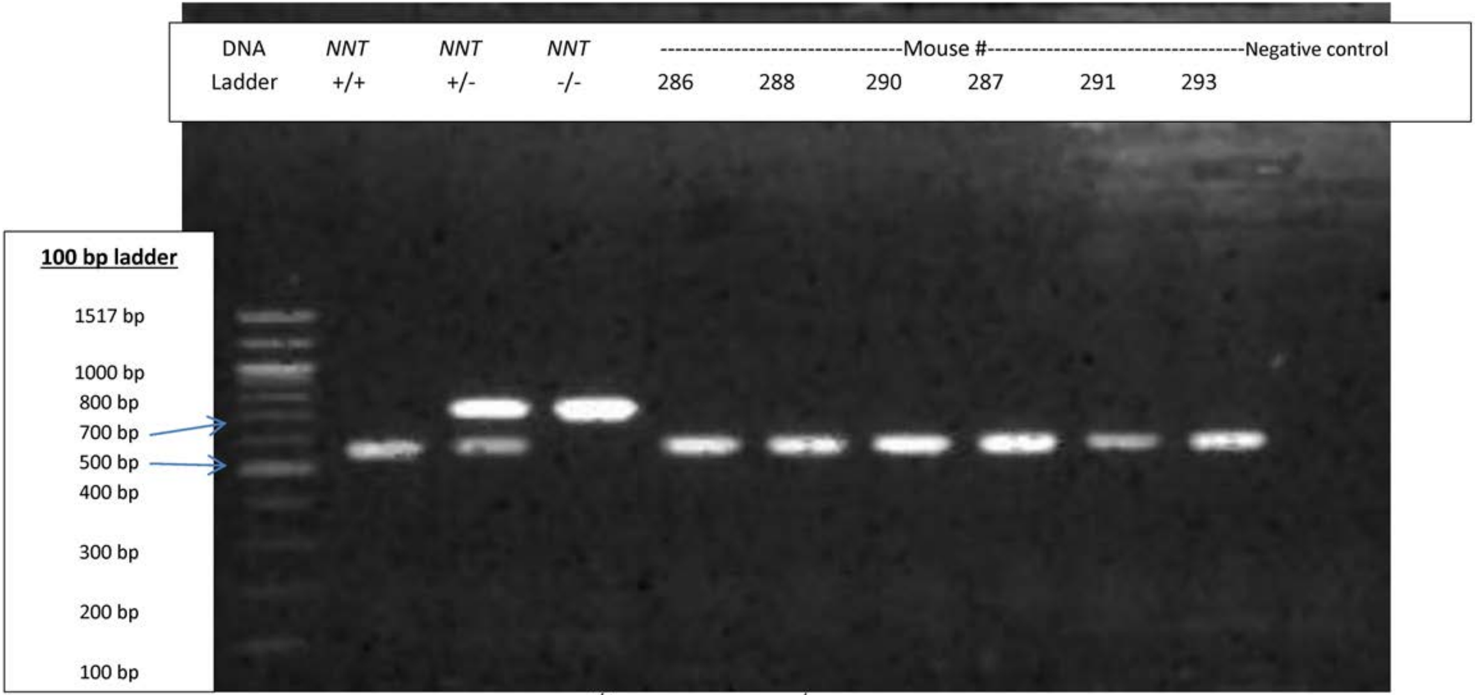

Expected Fragment Sizes: *NNT* $^{+/+}$ = 579 bp; *NNT* $^{-/-}$ = 743 bp.

Supplementary Figure S6
